# Biomimetic delivery of a STING agonist *via* tumor antigen-primed dendritic cell membrane nanovesicles for bladder cancer immunotherapy

**DOI:** 10.1016/j.mtbio.2026.103333

**Published:** 2026-06-11

**Authors:** Xianlu Zhang, Peng Xin, Yang Du, Ran Wang, Yutao Wang, Jianbin Bi, Yang Liu

**Affiliations:** a Department of Urology, The First Hospital of China Medical University, Shenyang, Liaoning, 110001, China; b Mater Research Institute, The University of Queensland, Brisbane, QLD, 4102, Australia; c Institute of Chemical Biology, Shenzhen Bay Laboratory, Shenzhen, 518132, China; d Department of Urology, Peking Union Medical Collage Hospital, Beijing, China

**Keywords:** Dendritic cell membrane nanovesicles, Bladder cancer, STING agonist, Tumor antigen priming, Biomimetic nanoplatform, Cancer immunotherapy

## Abstract

STING agonists hold considerable promise for cancer immunotherapy, but their therapeutic efficacy is often limited by poor cellular delivery, rapid systemic clearance, and insufficient activation of antigen-presenting cells. Here, we report an engineered dendritic cell membrane-derived nanovesicle platform (Ag-DCNV-STINGa) for biomimetic delivery of a STING agonist in bladder cancer immunotherapy. Generated from tumor antigen primed mature human monocyte-derived dendritic cell membranes, Ag-DCNV-STINGa preserves key antigen-presentation features and enables stable surface anchoring of 2′3′-cGAMP. Ag-DCNV-STINGa efficiently enhances STING agonist uptake by dendritic cells, promotes dendritic cell activation, and amplifies downstream T-cell-mediated antitumor immunity in vitro. In immunity humanized NSG mice, systemic administration of Ag-DCNV-STINGa exhibits favorable biosafety, suppresses tumor growth in subcutaneous and orthotopic bladder cancer models, and reduces lung metastatic burden. Mechanistically, Ag-DCNV-STINGa reshapes the tumor immune microenvironment by enhancing immune cell infiltration, sustaining interferon-associated immune activation, and promoting the formation of tertiary lymphoid structure-like immune aggregates. Together, these findings establish Ag-DCNV-STINGa as a biomimetic immunoengineering platform that integrates dendritic cell membrane functionality, tumor antigen information, and STING pathway activation to potentiate antitumor immunity. This strategy provides a promising approach for bladder cancer immunotherapy and may offer a broadly applicable framework for the delivery of innate immune agonists in cancer treatment.

## Graphical abstract

**Figure:**
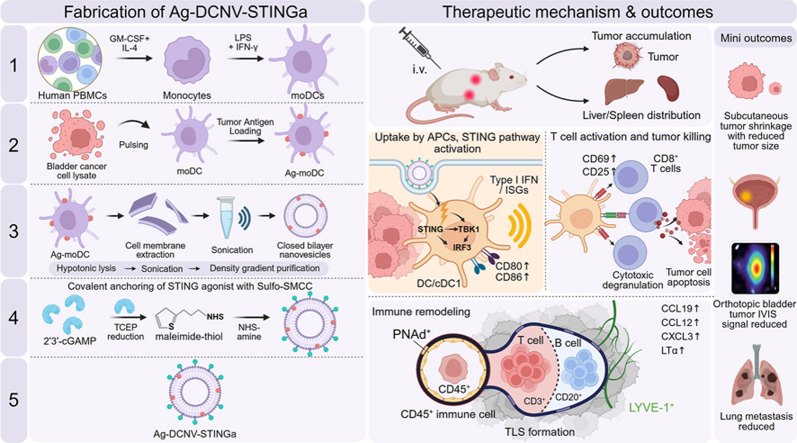

## 1 Introduction

Immune surveillance is a dynamic defense process through which the immune system recognizes and eliminates abnormal cells via coordinated innate and adaptive immune networks. When tumor cells evade immune recognition and remodel the immunosuppressive tumor microenvironment (TME), the capacity for tumor clearance and tissue homeostasis is progressively impaired, thereby driving disease progression [1]. In recent years, immunotherapeutic strategies such as chimeric antigen receptor (CAR) T-cell therapy, cancer vaccines, and immune checkpoint blockade (ICB) have developed rapidly and are now regarded as major strategy of treatment for multiple malignancies [[2], [3], [4]]. Immunotherapy is particularly important in bladder cancer therapy. From conventional intravesical BCG instillation to PD-1/PD-L1 blockade, these clinical applications show that bladder cancer is sensitive to immune therapies. However, the overall response rate remain limited because of an immunosuppressive TME and less effector cell infiltration [[5], [6], [7]].

Nanotechnology offers an engineering framework for cancer immunotherapy for their advantages in spatiotemporal delivery and signal integration. In particular, the cell membrane based biomimetic nanosystems, including cell membrane-coated nanoparticles and biomimetic membrane nanovesicles, have emerged as an important bridge between materials science and tumor immunology because of their intrinsic biocompatibility, preservation of membrane protein functions, and programmable drug-loading capacity [[8], [9], [10], [11]]. Compared with conventional nanoparticles, dendritic cell membranes carry major histocompatibility complex (MHC) molecules and co-stimulatory ligands on their outer surface. These features allow them to mimic the functional interface of antigen-presenting cells (APCs) and provide molecular basis for T cell priming [12]. At the same time, the membrane-biomimetic architecture improves in vivo stability and systemic tolerability, thereby offering a suitable carrier framework for the controlled delivery and amplification of immunostimulatory signals [13]. Moreover, if the tumor-derived antigens are preloaded on dendritic cells. The membrane nanovesicles may retain the tumor antigen-associated information, so that providing an antigen-presentation interface for further immune activation.

Among innate immune pathways, the cGAS-STING axis has attracted extensive attention because of its central role in sensing pathogen-associated and tumor-associated signals. STING agonists (STINGa) induce type I interferon production and the expression of downstream interferon-stimulated genes (ISGs), thereby promoting dendritic cell maturation, enhancing antigen cross-presentation, and amplifying CD8^+^ T cell-mediated antitumor immunity [[14], [15], [16]]. However, the in vivo application of free cyclic dinucleotide STING agonists, such as 2′3′-cGAMP, faces several major limitations [17]. Their highly hydrophilic and negatively charged nature leads to poor membrane permeability, while rapid systemic clearance, limited stability, and non-specific inflammatory exposure further restrict their therapeutic performance and clinical translation [18]. Therefore, the precise and efficient delivery of STING signals to APCs, while maintaining an acceptable safety profile, represents a key engineering challenge for improving the therapeutic window of STING-based immunotherapy. Recent studies have shown that nanoformulated or local delivery of STING agonists can substantially enhance immune activation and improve therapeutic outcomes, especially in bladder cancer, highlighting the concept that the mode of delivery critically determines both efficacy and safety [[19], [20], [21]].

Beyond the intensity of immune activation, another major factor of therapeutic outcome is whether the tumor immune landscape can evolve from scattered immune infiltration to an organized immune response. Tertiary lymphoid structures (TLSs) are ectopic lymphoid-like aggregates that arise in tumors or chronically inflamed tissues. They are characterized by distinct T-cell and B-cell zones, PNAd^+^ high endothelial venule (HEV)-mediated immune cell accumulation and associated lymphatic-like structures [22,23]. TLSs are frequently associated with stronger antitumor immunity and improved responses to immunotherapy, and are therefore increasingly recognized as both a biological marker and a potential amplifier of treatment response [24,25]. Accordingly, the induction of local chemokine programs, the recruitment of immune cells, and the formation of TLS/HEV-like structures may determine whether immunotherapy progresses from transient activation to sustained tumor control [26].

**Fig. 1:**
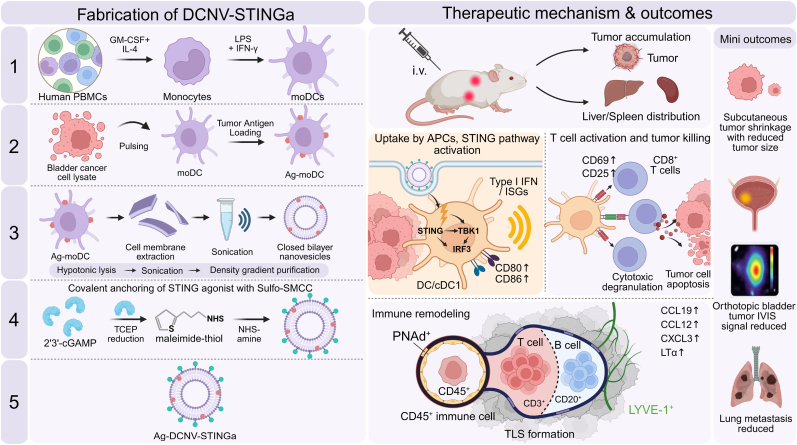
**Schematic illustration of the fabrication process and in vivo therapeutic mechanism of Ag-DCNV-STINGa.** The left panel shows the construction of Ag-DCNV-STINGa. Human PBMCs were differentiated into monocytes and further induced into moDCs in the presence of GM-CSF and IL-4, followed by maturation with LPS and IFN-γ. Mature moDCs were pulsed with bladder cancer cell lysate for tumor antigen loading. The moDCs loaded with cancer antigen were then subjected to hypotonic lysis to isolate cell membranes. The membrane fragments were physically reassembled into closed bilayer nanovesicles by sonication, followed by density gradient purification to obtain dendritic cell membrane nanovesicles. Subsequently, 2′3′-cGAMP was covalently anchored onto the vesicle surface using the heterobifunctional linker Sulfo-SMCC, involving TCEP-mediated reduction, maleimide-thiol reaction, and NHS-amine coupling with membrane proteins, to finally generate Ag-DCNV-STINGa. The right panel illustrates the in vivo trafficking and therapeutic outcomes of Ag-DCNV-STINGa. After intravenous administration, Ag-DCNV-STINGa showed detectable accumulation in tumors and distribution in reticuloendothelial organs. Following uptake by dendritic cells/cDC1-like antigen-presenting cells, Ag-DCNV-STINGa delivered the STING agonist and activated the STING-TBK1-RF3 signaling axis, inducing type I interferon/ISG responses while promoting DC maturation and upregulation of costimulatory molecules. Mature DCs further enhanced T-cell activation and promoted cytotoxic degranulation of CD8^+^ T cells. Meanwhile, the upregulation of tumor-localized chemokines promoted immune cell recruitment and spatial organization, accompanied by the appearance of PNAd^+^ high endothelial venule-like structures and LYVE-1^+^ lymphatic-associated structures, ultimately driving tertiary lymphoid structure (TLS) formation with distinct T-cell and B-cell zones. These processes collectively resulted in reduced subcutaneous tumor burden, decreased IVIS signal in orthotopic bladder tumors, and reduced lung metastatic load.

Based on this circumstance, we developed and validated an engineered dendritic cell membrane nanovesicle platform, termed Ag-DCNV-STINGa (antigen-primed dendritic cell membrane nanovesicles carrying STING agonist), as shown in Fig. 1. In this system, mature moDC membranes derived from human PBMCs were first pulsed with bladder cancer cell lysates. So that their membranes were isolated and physically reassembled into the membrane-derived nanovesicles. The STING agonist 2′3′-cGAMP was then stably anchored onto the vesicle suface through chemical conjugation. This design enabled efficient delivery to antigen-presenting cells and amplification of STING signaling. In vitro, Ag-DCNV-STINGa markedly enhanced T-cell activation, promoted inflammatory and chemotactic cytokine secretion, and increased tumor-cell killing in PBMC-bladder cancer co-culture systems. In Hu-PBMC reconstituted NSG mice, Ag-DCNV-STINGa produced consistent antitumor effects in subcutaneous, orthotopic, and lung metastasis models. Moreover, treatment was associated with clear remodeling of the local immune microenvironment, including increased CD8^+^ T-cell infiltration and effector function, a higher proportion of mature dendritic cells, upregulation of chemokines such as CCL19 and CCL21 and ISGs, and the appearance of TLS- and PNAd^+^ HEV-related structures. Overall, this study presents a delivery-engineering strategy that integrates STING signaling into a dendritic cell membrane biomimetic carrier. These provides both theoretical basis and experimental support for the development of safer and more effective immunotherapies for bladder cancer with the capacity to induce organized immune remodeling.

## 2 Methods

### 2.1 Materials and reagents

2′3′-cGAMP (STING agonist), its fluorescently labeled derivative (Cy5-STINGa), and the heterobifunctional crosslinker Sulfo-SMCC were purchased from Dalian Meilun Biotechnology (Dalian, China). The DiO membrane-labeling kit, DiR near-infrared dye, CCK-8 kit, Calcein-AM/PI live/dead staining kit, and Ficoll solution for human peripheral blood lymphocyte isolation were purchased from Beyotime Biotechnology (Shanghai, China). ELISA kits for CXCL10 (AB83700), IL-2 (AB270883), IFN-γ (AB46025), and TNF-α (AB181421) were obtained from Abcam. TRIzol reagent, reverse transcription/RT-qPCR reagents, RIPA lysis buffer, protease/phosphatase inhibitors, the BCA protein assay kit, PVDF membranes, and ECL substrate were used according to the manufacturers’ instructions. Unless otherwise specified, all centrifugation steps were performed at 4 °C.

### 2.2 Animals

Six-to eight-week-old NSG immunodeficient mice (BEIJING HFK BIOSCIENCE, China) were used for Hu-PBMC humanized immune reconstitution and for the establishment of subcutaneous, orthotopic, and lung metastasis bladder cancer models [27]. Immunodeficient mice were also used for in vivo safety evaluation and biodistribution studies. Experimental and control mice were co-housed and distributed across different cages. Animals were maintained at approximately 25 °C with 50 ± 5% humidity under a 12-h light/dark cycle and had free access to standard chow and water. Humane endpoints included tumor burden exceeding 10% of body weight, body weight loss >20%, tumor ulceration, or persistent self-injury. Mice were euthanized by cervical dislocation under deep anesthesia. All procedures were conducted in accordance with the guidelines of the Animal Ethics Committee of China Medical University. This study complied with all relevant ethical regulations. All animal experiments and clinical sample collection procedures were conducted in accordance with the Guide for the Care and Use of Laboratory Animals and the requirements for informed consent, and were approved by the Animal Ethics Committee of China Medical University and the Clinical Ethics Committee of the First Hospital of China Medical University.

### 2.3 Cell lines and culture

The human bladder cancer cell lines UM-UC-3 (catalog no. TCHu217) and T24 (catalog no. SCSP-536) were obtained from the Cell Bank of the Chinese Academy of Sciences. The luciferase-labeled T24-Luc cell line (catalog no. LZQ0068) was purchased from Zhong Qiao Xin Zhou Biotechnology (Shanghai, China). Cells were cultured in MEM or RPMI-1640 medium (Gibco) supplemented with 10% fetal bovine serum (FBS; Gibco), 100 U mL^−1^ penicillin, and 100 μg mL^−1^ streptomycin (Invitrogen) at 37 °C in 5% CO_2_. All cells were tested and confirmed to be mycoplasma-free before use.

### 2.4 Hu-PBMC humanized immune reconstruction

Peripheral blood mononuclear cells (PBMCs) and monocyte-derived dendritic cells (moDCs) were obtained from at least three independent healthy donors. When used in the same immune activation experiment, PBMCs and moDCs were derived from the same donor whenever possible to reduce donor-specific variation. PBMCs were isolated by Ficoll density-gradient centrifugation. All donors provided written informed consent, and the protocol was approved by the institutional ethics committee. Whole blood was processed according to the manufacturer's instructions, and the isolated PBMCs were washed with PBS two to three times to remove platelets. Cell viability was determined by trypan blue exclusion, and only cell suspensions with viability >90% were used for injection. Cells were resuspended in sterile PBS. For humanized immune reconstruction, NSG mice received 1 × 10^7^ PBMCs in 100 μL PBS via tail vein injection. Tumor inoculation and treatment were initiated 7 days later, or the extent of human immune cell engraftment was assessed as required. To maintain human immune cell activity and persistence, recombinant human IL-2 (5000 IU per mouse) was administered intraperitoneally every 2 days starting on day 2 after PBMC injection and continued until the experimental endpoint. In vitro experiments were performed with at least three independent biological replicates derived from healthy donors. In vivo experiments report the number of mice per group. Technical replicates, such as ELISA measurements, are also indicated in the figure legends.

### 2.5 Induction and maturation of moDCs

Monocytes were obtained from PBMCs by an adherence method. Briefly, PBMCs were palted into culture dishes and incubated for 2 h. Then we removed non-adherent cells, and the adherent monocytes were cultured in medium containing GM-CSF and IL-4 to induce differentiation. Cells were maintained for 6 days, the culture medium was partially replaced and cytokine was added every other day. To obtain mature moDCs with a stable phenotype, LPS (100 ng/mL) and IFN-γ (1000 U/mL) were added during the last stage of differentiation.

### 2.6 Tumor antigen pulsing of mature moDCs

To incorporate bladder cancer-derived antigen information into dendritic cell membrane nanovesicles, mature moDCs were pulsed with bladder cancer cell lysates. Briefly, T24 cells were collected at 80-90% confluence, washed twice with cold PBS, resuspended in sterile PBS. Tumor lysates were prepared by repeated freeze-thaw cycles (five cycles of freezing at −80 °C and thawing at 37 °C). They were then centrifugation at 12,000g for 10 min at 4 °C to remove large cellular debris. The supernatant containing soluble tumor-derived antigen components was collected, the total protein concentration was determined using the BCA assay. Mature moDCs were then incubated with tumor lysate for 12 h. After incubation, cells were washed three times with cold PBS to remove unbound lysate components.

### 2.7 Preparation of dendritic cell membrane nanovesicles

Membranes were isolated from tumor antigen-pulsed moDCs as described above. They were washed three times with PBS to remove serum proteins, resuspended in PBS with 5 mL, and sonicated on ice (150 W, 10 min). The sequential differential centrifugation: 2000 rpm for 10 min to remove unbroken cells and nuclei, 5000 rpm for 10 min to remove mitochondria and large organelle fragments, and 15,000 rpm for 60 min to pellet membrane-rich fractions. The membrane pellet was collected, resuspended in PBS, and filtered through a 200-nm membrane to obtain purified DC membrane nanovesicles (DCNVs) with a uniform size distribution. The final product was stored at 4 °C for short-term use or at −80 °C for long-term storage.

### 2.8 STING agonist loading and validation

2′3′-cGAMP was used as the STING agonist, and the in vivo dose was set at 20 μg per mouse for each administration. To attach STINGa onto the surface of DCNVs, Sulfo-SMCC was used as the coupling reagent. Thiolated 2′3′-cGAMP was dissolved in PBS at 100 μM and reduced with TCEP (1 mol/L) at room temperature for 30 min with gentle mixing. Sulfo-SMCC was then added and incubated for another 30 min. After that, the mixture was washed three times with PBS to remove excess TCEP and unreacted Sulfo-SMCC. The resulting intermediate was then incubated with DCNVs at room temperature for 1 h. The product was purified and washed using a 100 kDa molecular weight cut-off centrifugal filter to remove free STINGa and small molecular weight. For validation of surface conjugation, DCNVs were labeled with DiO, and Cy5-labeled STINGa was incubated with DCNVs in the presence or absence of Sulfo-SMCC. DiO (green) and Cy5 (red) signals were acquired by confocal microscopy and analyzed for colocalization to verify stable surface attachment of STINGa. The 2′3′-cGAMP equivalent amount in Ag-DCNV-STINGa was estimated based on the recovered STING agonist signal after removal of free unconjugated 2′3′-cGAMP using centrifugal filtration.

### 2.9 Physicochemical and biological characterization of Ag-DCNV-STINGa

Samples were deposited onto copper grids, negatively stained with uranyl acetate, and imaged by transmission electron microscopy (TEM; JEM-1400 Flash) to assess vesicle morphology, size, and membrane integrity. Dynamic light scattering (DLS) was used to determine hydrodynamic diameter and polydispersity index (PDI), and zeta potential was measured to evaluate colloidal stability. Equal amounts of DCNVs and mature moDC lysates were analyzed by SDS-PAGE to assess overall protein profiles. Functional membrane proteins were further examined by Western blotting. Briefly, proteins were separated by SDS-PAGE, transferred onto PVDF membranes, blocked with 5% skim milk for 1 h, and incubated overnight at 4 °C with primary antibodies against Na/K ATPase (Abcam, ab254025, 1:1000), MHC I (Abcam, ab225636, 1:1000), MHC II (Abcam, ab55152, 1:1000), CD11c (Abcam, ab52632, 1:1000), and CD86 (Abcam, ab220188, 1:1000). Membranes were then incubated with HRP-conjugated secondary antibodies for 1 h at room temperature, and signals were visualized using ECL substrate. This analysis was used to verify retention of antigen-presentation- and co-stimulation-related membrane proteins. Raw scans of all blots are provided in the source data. Surface MHC I was further evaluated by flow cytometry using AF647-conjugated anti-MHC I antibody. Similarly, surface EGFR was also evaluated by flow cytometry using PE-conjugated anti-EGFR antibody. Stability during storage was assessed by keeping DCNVs at 4 °C or 37 °C for 1 month and measuring particle size and PDI at different time points.

### 2.10 Cellular uptake studies

For bio-TEM analysis, dendritic cells were incubated with Ag-DCNV-STINGa for 12 h and then fixed with 2.5% glutaraldehyde. Samples were washed three times with 0.1 M phosphate buffer for 10 min each, post-fixed with 10 g/L osmium tetroxide at 4 °C for 1 h, and washed with distilled water three times. Samples were dehydrated through graded ethanol (30%, 50%, 70%, 90%, and 100%) for 5 min each, followed by replacement with 100% propylene oxide twice for 5 min each. Cells were infiltrated with epoxy resin/propylene oxide (1:1) for 1 h, then with pure epoxy resin for 1 h, embedded in molds, and polymerized at 60 °C for 24 h. Ultrathin sections (70 nm) were cut using an ultramicrotome (Leica UC7), mounted on copper grids, stained with 30 g/L uranyl acetate for 20 min and 26 g/L lead citrate for 5 min, washed with distilled water, air-dried, and imaged by TEM (JEM-1400 Flash) at 120 kV to visualize nanovesicles within endocytic vesicle-like structures. For flow cytometric uptake analysis, cDC1 cells were incubated with either free Cy5-2′3′-cGAMP or DCNV-delivered Cy5-2′3′-cGAMP. At indicated time points, cells were collected, washed with PBS to remove extracellular probe, and analyzed by flow cytometry. The percentage of Cy5-positive cells and the mean fluorescence intensity (MFI) were recorded to compare cellular uptake under free and DCNV-mediated delivery conditions.

### 2.11 In vitro immune activation and tumor killing

Human PBMCs were co-cultured with bladder cancer cells. UM-UC-3 and T24 cells were used as target cells and were seeded in advance to allow adherence and growth to an appropriate density. PBMCs were then added to establish immune cell-tumor cell co-cultures. Cultures were maintained at 37 °C in 5% CO_2_, and the number of PBMCs added was adjusted according to the desired effector-to-target ratio (E:T) for subsequent assays of tumor cell viability, live/dead staining, cytokine secretion, and immune phenotyping. Treatment groups included PBS, empty DCNVs, DCNV-STINGa (without antigen loading), and Ag-DCNV-STINGa. Co-culture was continued for 24 h at E:T ratios of 5:1, 10:1, and 20:1. Tumor cell viability was then assessed using the CCK-8 assay according to the manufacturer's instructions. Absorbance was measured to calculate relative tumor cell viability normalized to the control group. For live/dead staining, culture medium was removed after co-culture, cells were stained with Calcein-AM/PI according to the manufacturer's protocol, imaged using an inverted fluorescence microscope, and quantified with ImageJ software.

### 2.12 ELISA

After PBMC-tumor cell co-culture, supernatants were collected and centrifuged at 4 °C to remove cells and debris. Aliquots were stored at −80 °C when necessary to avoid repeated freeze-thaw cycles. Levels of CXCL10, IL-2, IFN-γ, and TNF-α were measured using commercial ELISA kits according to the manufacturers’ instructions. Briefly, serially diluted standards were used to generate standard curves, samples and standards were added to the coated plates, followed by incubation, washing, and sequential addition of detection antibody and enzyme conjugate. After color development, the reaction was stopped and absorbance was read at 450 nm. Concentrations were calculated from the standard curves and corrected for dilution factors. Five technical replicates were included for each sample, and blank wells and inter-plate controls were used for quality control.

### 2.13 Flow cytometry

For in vitro flow cytometry, cells from PBMC-tumor co-cultures were collected, washed with PBS, filtered through a cell strainer, and prepared as single-cell suspensions. Surface staining was performed for 50 min at 4 °C in the dark, followed by PBS washing and acquisition on a flow cytometer. T-cell activation markers (CD69 and CD25) and APC maturation markers (CD80 and CD86) were analyzed. Unstained controls, isotype controls, and single-color compensation controls were included. The antibody panel used was as follows: PerCP-Cy5.5 mouse anti-human CD45 (Absin, abs1840504), FITC mouse anti-human CD3 (Absin, abs1840001), BV421 mouse anti-human CD69 (BD Pharmingen, 562884), BV605 mouse anti-human CD25 (BD Pharmingen, 567572), APC mouse anti-human CD11c (BD Pharmingen, 560895), FITC mouse anti-human CD80 (BD Pharmingen, 560926), and PE mouse anti-human CD86 (BD Pharmingen, 560957). For in vivo immune profiling, tumors were excised at the endpoint, minced, and enzymatically digested to prepare single-cell suspensions. Cells were stained for 50 min and analyzed for the proportion of CD8^+^ T cells, CD8^+^ T-cell degranulation (CD107a), the proportion of CD20^+^ cells among CD45^+^ immune cells, and the proportion of CD80^+^CD86^+^ double-positive cells among CD11c^+^ dendritic cells. The antibody panel used was: PerCP-Cy5.5 mouse anti-human CD45 (Absin, abs1840504), FITC mouse anti-human CD3 (Absin, abs1840001), PE mouse anti-human CD4 (BD Pharmingen, 566680), BV650 mouse anti-human CD8 (BD Pharmingen, 563822), PE mouse anti-human CD20 (BD Pharmingen, 561174), APC mouse anti-human CD11c (BD Pharmingen, 560895), FITC mouse anti-human CD80 (BD Pharmingen, 560926), and PE mouse anti-human CD86 (BD Pharmingen, 560957). To verify successful human immune reconstitution in NYG mice, spleens were aseptically collected after humanization, gently dissociated through a filter, and centrifuged for 5 min. After washing, cell pellets were resuspended in red blood cell lysis buffer for 5 min, prepared as single-cell suspensions, stained for 50 min, washed with PBS, and analyzed for human immune cell subsets using PerCP-Cy5.5 mouse anti-human CD45, FITC mouse anti-human CD3, PE mouse anti-human CD4, BV650 mouse anti-human CD8, and APC mouse anti-human CD11c. Flow cytometry data were analyzed using FlowJo software.

### 2.14 Western blotting

Cells were harvested at the indicated time points, washed with ice-cold PBS, and lysed on ice in RIPA buffer containing protease and phosphatase inhibitors. Lysates were centrifuged at 12,000 × g for 10 min at 4 °C, and the supernatants were collected. Tumor tissues were minced on ice, homogenized in tissue lysis buffer, and centrifuged, and the supernatants were collected in the same manner. Protein concentrations were determined by BCA assay, and equal amounts of protein were loaded. Samples were mixed with SDS loading buffer, denatured, separated by SDS-PAGE, and transferred to PVDF membranes. Membranes were blocked with 5% skim milk for 1 h at room temperature and incubated overnight at 4 °C with primary antibodies against TBK1 (Abcam, ab40676, 1:1000), p-TBK1 (Abcam, ab109272, 1:1000), IRF3 (Abcam, ab68481, 1:1000), p-IRF3 (Abcam, ab76493, 1:1000), ISG15 (Abcam, ab285367, 1:1000), MX1 (Abcam, ab95926, 1:1000), and IFIT1 (Abcam, ab305301, 1:1000). After washing with TBST, membranes were incubated with HRP-conjugated secondary antibodies for 1 h at room temperature. Bands were detected using ECL and quantified by densitometry with ImageJ.

### 2.15 In vitro and in vivo safety assessment and biodistribution

UM-UC-3 cells, normal bladder epithelial cells, HEK293 cells, and L02 hepatocytes were seeded in 96-well plates and incubated with DCNV-STINGa, followed by CCK-8 assay to evaluate direct material-related cytotoxicity. Fresh anticoagulated human blood was centrifuged at 800 × g to collect red blood cells (RBCs), which were washed three times with PBS and prepared as RBC suspensions. Hemolysis was evaluated after incubation with different concentrations of Ag-DCNV-STINGa. For in vivo safety studies, immunodeficient mice received multiple tail vein injections of DCNVs at different doses or PBS, and body weight was monitored continuously. Blood routine parameters and serum biochemistry were measured at the safety dose, and major organs were collected for H&E staining. For biodistribution studies, Ag-DCNV-STINGa was labeled with DiR and administered intravenously. Whole-body in vivo imaging was performed at different time points, and major organs were excised at 24 h for ex vivo IVIS imaging and fluorescence quantification.

### 2.16 In vivo antitumor studies

Ag-DCNV-STINGa was administered by tail vein injection at a dose of 250 μg vesicle protein per mouse per dose, corresponding to approximately 5 μg 2′3′-cGAMP equivalent per mouse. The free STINGa control group received 20 μg 2′3′-cGAMP per mouse per dose. Treatments were given every 3 days for a total of four injections (q3d × 4). The therapeutic dose of Ag-DCNV-STINGa was selected based on preliminary tolerability studies and optimization experiments to achieve maximal antitumor efficacy while minimizing toxicity. The dose used was 250 μg vesicle protein per mouse, corresponding to approximately 5 μg 2′3′-cGAMP equivalent. For the subcutaneous model, Hu-PBMC reconstituted mice were inoculated subcutaneously with T24 or UM-UC-3 bladder cancer cells. After tumor formation, mice were randomized and treated according to the q3d × 4 schedule. Tumor length and width were measured every 2 days, and tumor volume was calculated. For the orthotopic model, T24-Luc cells were inoculated into the bladder wall of NSG mice under laparotomy after humanized immune reconstruction. Tumor burden was monitored by IVIS, and tumors were weighed at the endpoint. For the lung metastasis model, T24-Luc cells were injected via the tail vein at the time of subcutaneous tumor establishment to induce pulmonary metastasis, followed by humanized immune reconstruction and treatment. At the endpoint, lungs were subjected to IVIS imaging, Bouin's fixation, metastatic nodule counting, and H&E staining. Mice were randomly assigned to treatment groups. Tumor measurements and histology quantification were performed by investigators blinded to the treatment groups.

### 2.17 Tumor tissue RT-qPCR and immunofluorescence

Total RNA was extracted from tumor tissues using TRIzol and reverse-transcribed into cDNA. qPCR was performed to detect the expression of chemokines, including CXCL3, CCL19, and CCL21. Relative expression levels were normalized to internal control genes and calculated using the 2^-ΔΔCt method. For immunofluorescence analysis, tumors were embedded and sectioned. Sections were stained for LYVE-1 alone, for CD3/CD20, or for PNAd/CD45, according to the intended analysis of lymphatic-like structures, T/B-cell zonation, and HEV-related structures.

### 2.18 Statistical analysis

Data are presented as mean ± standard deviation (SD). Comparisons between two groups were performed using Student's t-test. Multiple-group comparisons were performed using one-way ANOVA followed by post hoc testing. Statistical analyses were conducted using GraphPad Prism 10.0, and P < 0.05 was considered statistically significant.

## 3 Results

### 3.1 Preparation, characterization and intracellular uptake of dendritic cell membrane nanovesicles

**Fig. 2:**
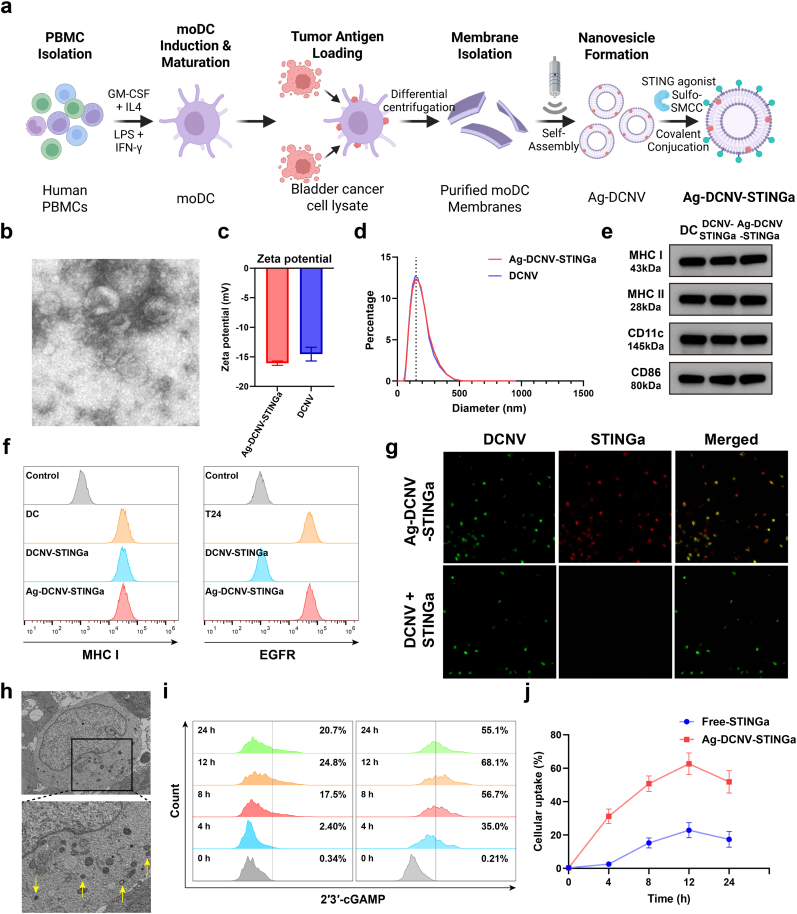
**Preparation, characterization, and cellular uptake of dendritic cell membrane nanovesicles.** (a) Schematic illustration of DCNV preparation. Cell membranes were isolated from human PBMC-derived mature monocyte-derived dendritic cells (moDCs) and were then loaded with cancer antigen. Membrane fractions were obtained by hypotonic lysis, followed by sonication-induced reassembly into closed bilayer vesicles and multistep density gradient centrifugation, getting membrane-derived nanovesicles. (b) Transmission electron microscopy (TEM) image of DCNVs, showing spherical or near-spherical vesicles with a clear bilayer membrane structure. (c,d) Physicochemical characterization of DCNVs: zeta potential (c) of approximately −15 to −20 mV and dynamic light scattering (DLS) analysis showing an average particle size of approximately 100–150 nm with PDI <0.2 (d). (e) Western blot analysis of membrane proteins in mature moDC lysates and DCNVs, showing stable retention of antigen-presentation-related molecules (MHC I and MHC II) and costimulatory molecules (CD11c and CD86) in DCNVs. (f) Flow cytometric analysis of MHC I (Left) and EGFR (Right) on the DCNV surface. (g) Validation of covalent anchoring of the STING agonist (STINGa) on DCNVs. DiO-labeled DCNVs (green) and Cy5-labeled STINGa (red) showed marked colocalization in the presence of Sulfo-SMCC, whereas colocalization was substantially reduced in the absence of the linker. (h) Bio-transmission electron microscopy (bio-TEM) showing effective internalization of Ag-DC-NVs-STINGa by dendritic cells, with vesicles localized within endocytic vesicle-like structures in the cytoplasm after 12 h treatment. (i,j) Quantification of STINGa uptake by cDC1 cells. Flow cytometric analysis showed that uptake of free fluorescently labeled 2′3′-cGAMP by cDC1 cells was low, with 24.8 ± 3.36% positive cells after 12 h incubation, whereas DC-NV-mediated delivery significantly enhanced uptake to 68.1 ± 6.11%.

To construct an immunoengineered nanoplatform, cell membranes were collected from human PBMC-derived mature monocyte derived dendritic cells (moDCs). The moDCs were then loaded with bladder cancer cell antigens. The dendritic cell membrane nanovesicles (DCNVs) were prepared by a physical reassembly process. Membrane fractions were obtained after cell disruption, and membrane fragments were reassembled into closed bilayer vesicles by sonication. The multistep density-gradient purification was then used to remove organelle components and soluble proteins (Fig. 2a). TEM showed that the nanovesicles showed regular spherical or near-spherical morphology with clear boundaries and a typical bilayer membrane structure (Fig. 2b). The zeta potential of DCNVs was approximately −15mV (Fig. 2e), similar to the charge characteristics of cell membrane-derived nanovesicles. Dynamic light scattering (DLS) showed an average particle size of approximately 120 nm, the PDI <0.2 (Fig. 2d). Western blotting showed that DCNVs retained membrane protein similar to the original moDCs. Antigen presentation related molecules, including MHC I and MHC II, as well as the co-stimulatory molecules CD11c and CD86, were stably preserved. These indicating that the preparation process did not loss key structures involved in antigen presentation and immune co-stimulation (Fig. 2e). Flow cytometric analysis further showed retention of antigen presentation related molecules on the vesicle surface. Compared with the unstained control, AF647-labeled anti-MHC I staining demonstrated a markedly increased MHC I signal on Ag-DCNVs-STINGa. Similarly, PE-labeled anti-EGFR staining demonstrated a markedly increased EGFR signal on Ag-DCNVs-STINGa compared with DCNV-STINGa. Suggesting that Ag-DCNVs-STINGa preserved key molecular components required for antigen presentation (Fig. 2f).

To verify the conjugation of STINGa, the vesicle membrane was labeled with DiO and Cy5-labeled STINGa was incubated with DCNVs in the presence or absence of Sulfo-SMCC. Confocal images showed clear colocalization of the green DiO signal and the red Cy5 signal when Sulfo-SMCC was present, whereas colocalization was substantially weaker without the crosslinker, indicating that STINGa was stably conjugated to the vesicle surface rather than merely adsorbed (Fig. 2g). Stability studies showed no obvious changes in particle size or functional proteins after storage at 4 °C or 37 °C for 1 week, indicating good colloidal stability and acceptable short-term storage stability (Figs. S1 and S2).

To evaluate the cellular uptake of Ag-DCNV-STINGa, bio-TEM showed that after 12h, the nanovesicles were localized within endocytic vesicle-like structures in the cytoplasm of dendritic cells, indicating efficient cellular internalization (Fig. 2h). The delivery efficiency of the STING agonist was also quantified by flow cytometry. Free fluorescently labeled 2′3′-cGAMP showed limited uptake in cDC1 cells and increased only modestly over time, reaching 24.8 ± 3.36% positive cells after 12 h of incubation (Fig. 2i and j). In contrast, DCNV-mediated delivery markedly enhanced 2′3′-cGAMP uptake, with approximately 68.1 ± 6.11% of cDC1 cells being positive (Fig. 2i and j), indicating that the membrane nanovesicles substantially improved the entry of the STING agonist into antigen-presenting cells. Taken together, these results demonstrate the successful construction of an engineered nanovesicle platform derived from dendritic cell membranes. The vesicles showed a uniform size distribution, a stable surface charge, and preserved membrane protein components. Key molecules involved in antigen presentation and co-stimulation remained detectable after vesicle preparation. STING agonist were also be coupled on the surface by chemical conjugation. In addition, DCNVs promoted dendritic cell uptake more efficiently than free STINGa, supporting the potential of this system for efficient delivery of innate immune activators and subsequent induction of antitumor immunity.

### 3.2 DC nanovesicles induce immune activation and promote bladder cancer cell killing

**Fig. 3:**
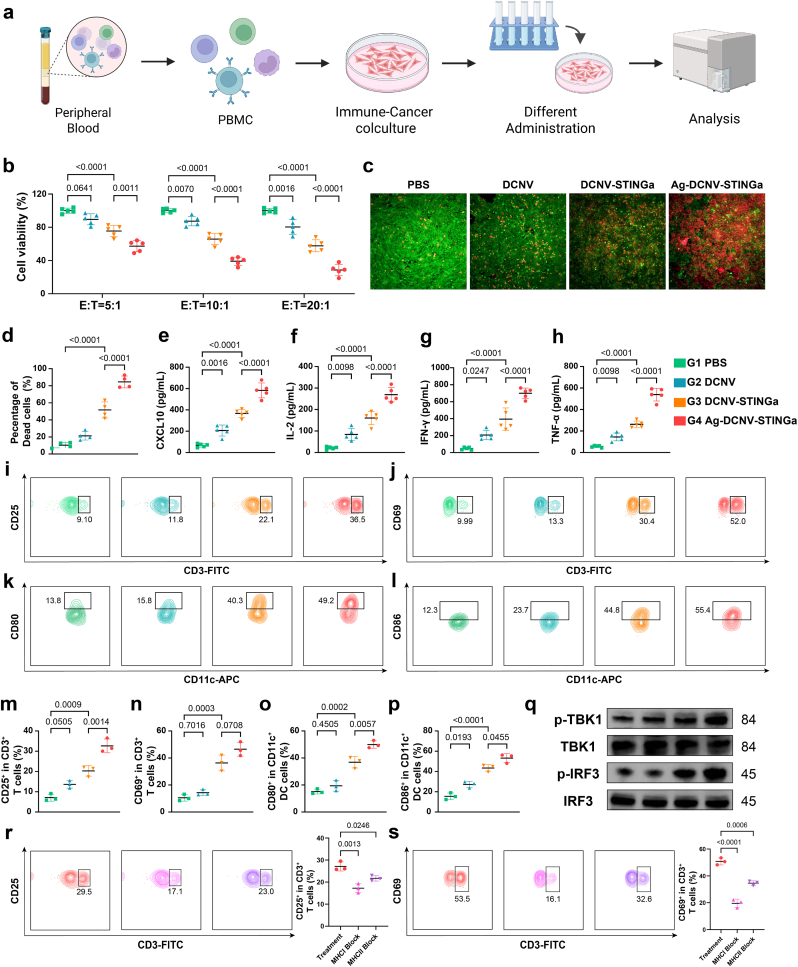
**Ag-DCNV–STINGa induces immune activation in vitro and enhances bladder cancer cell killing.** (a) Schematic illustration of the coculture system of human PBMCs and bladder cancer cells (24 h). (b) CCK-8 analysis of tumor cell viability at different effector-to-target ratios (E:T = 5:1, 10:1, and 20:1). (c, d) Representative live/dead fluorescence staining images and quantitative analysis. (e-h) ELISA analysis of cytokine/chemokine levels in coculture supernatants: CXCL10 (e), IL-2 (f), IFN-γ (g), and TNF-α (h). (i,j) Representative flow cytometric plots and statistical analysis of T-cell activation markers CD69 (i, m) and CD25 (j, n) in different treatment groups. (k,l) Representative flow cytometric plots; (o,p) corresponding quantitative analysis of dendritic cell maturation/costimulatory markers CD80 (k,o) and CD86 (l,p) in antigen-presenting cell populations. (q) Western blot analysis of phosphorylated STING pathway-related signaling molecules in mature moDCs after short-term stimulation, including p-TBK1/TBK1 and p-IRF3/IRF3. (r,s) Representative flow cytometric plots and statistical analysis of T-cell activation markers CD25 (r) and CD69 (s), comparing the treatment of Ag-DCNV-STINGa and the blockage of MHCI and MHCII.

To evaluate the immunological activity of Ag-DCNV-STINGa, we first established co-culture systems of human PBMCs with bladder cancer cell line T24 (Fig. 3a). PBMCs and bladder cancer cells were co-cultured for 24 h and treated with PBS, empty DCNVs, DCNV-STINGa, or Ag-DCNV-STINGa to compare antitumor immune effects under different conditions. At different effector-to-target ratios (E:T = 5:1, 10:1, and 20:1), tumor cell viability was measured by CCK-8. In T24 co-cultures, the PBS group showed the highest tumor cell viability, indicating that PBMCs alone had limited intrinsic cytotoxicity against tumor cells. Empty DCNVs caused a mild reduction in viability, suggesting that co-stimulatory molecules on the dendritic cell membrane provided a certain degree of immune enhancement. Ag-DCNV-STINGa further increased tumor cell killing. Notably, Ag-DCNV-STINGa produced the strongest cytotoxic effect at all E:T ratios, and the killing effect increased with the effector-to-target ratio (Fig. 3b). Live/dead staining was consistent with the CCK-8 results, showing a marked increase in red dead cells after Ag-DCNV-STINGa treatment, whereas green live cells predominated in the PBS group (Fig. 3c and d). Similar results were reproduced in another bladder cancer cell line UM-UC-3, indicating that the immune killing effect was reproducible rather than cell-line specific (Figs. S3 and S4).

To further characterize the type of immune response induced, we measured cytokines and chemokines in the co-culture supernatants by ELISA. The levels of CXCL10, IL-2, IFN-γ, and TNF-α were lowest in the PBS group, modestly increased in the empty DCNV group, further elevated in the DCNV-STINGa group, and reached the highest levels in the Ag-DCNV-STINGa group (Fig. 3e–h). CXCL10 suggested activation of STING-induced type I interferon-related chemotactic signaling, whereas IL-2 and IFN-γ reflected enhanced T-cell activation and a Th1-skewed immune response. Increased TNF-α was consistent with strengthened cytotoxic immunity, indicating that the nanovesicle system induced a predominantly cytotoxic T-cell oriented antitumor response. Flow cytometric analysis of immune phenotypes further showed that, compared with PBS, Ag-DCNV-STINGa markedly increased the expression of the early T-cell activation marker CD69 and the IL-2 receptor α-chain CD25 (Fig. 3i,j,m,n), indicating efficient T-cell activation. In addition, the expression of the co-stimulatory molecules CD80 and CD86 was clearly upregulated in antigen-presenting cell populations (Fig. 3k,l,o,p), indicating activation and maturation of dendritic cells. The same results were also observed in the co-culture system between immune cells and UM-UC-3 cells (Figs. S5 and S6). Isolated human moDCs were treated with PBS, DCNV, free STINGa, DCNV-STINGa, or Ag-DCNV-STINGa for 24 h. Flow cytometry analysis showed that Ag-DCNV-STINGa markedly increased CD80, CD86, and MHC I expression (Fig. S7). This provides direct evidence that Ag-DCNV-STINGa promotes dendritic cell maturation. Together, these findings suggest that Ag-DCNV-STINGa can simultaneously act on antigen-presenting cells and effector T cells to produce coordinated immune activation.

To determine whether immune activation was driven by the canonical STING pathway rather than by nonspecific stimulation from membrane co-stimulatory molecules alone, we examined phosphorylation of key STING downstream signaling proteins in isolated dendritic cells (Fig. 3q). PBMC-derived mature moDCs were treated with PBS, empty DCNVs, DCNV-STINGa, or Ag-DCNV-STINGa for a short time and then analyzed by Western blotting. Compared with PBS and empty DCNVs, Ag-DCNV-STINGa significantly increased the phosphorylation of TBK1 (p-TBK1) and IRF3 (p-IRF3), whereas total TBK1 and total IRF3 levels remained unchanged, indicating specific activation of the STING-TBK1-IRF3 axis. To avoid the complexity of mixed signals and dilution of phospho-signals in tumor-immune cell co-cultures, pathway analysis was not performed using whole co-culture lysates. Together with the marked increases in IFN-γ, IL-2, and CXCL10 and the enhancement of T-cell activation and tumor killing observed in co-cultures, these data further support that Ag-DCNV-STINGa effectively activates STING signaling in dendritic cells and drives downstream immune effector output. To confirm that Ag-DCNV-STINGa-induced immune activation and tumor killing depend on STING signaling, PBMC–bladder co-cultures were treated with Ag-DCNV-STINGa in the presence or absence of the STING inhibitor H-151. STING inhibition reduced CXCL10, IFN-γ, and IL-2 secretion and attenuated tumor cell killing (Fig. S8), indicating that the immune activation is STING-dependent.

In addition, we performed the MHC I and MHC II blockage assay to verify the antitumor immunity of Ag-DCNV-STINGa is relied on its cancer antigen and through classic antigen presenting pathway mediated by MHC. Flow cytometric analysis showed that, compared with the Ag-DCNV-STINGa group, blockade of MHC I markedly impaired immune cell activation, as evidenced by a significant reduction in CD25 and CD69 expression. MHC II blockade also decreased the expression of these activation markers, although the magnitude of inhibition was less pronounced than that observed after MHC I blockade (Fig. 3r and s). ELISA results showed a similar pattern. The levels of IL-2 and IFN-γ were substantially reduced in the MHC I blocking group and were also decreased in the MHC II blocking group, but to a lesser extent (Figs. S9 and S10). Consistent with these findings, the CCK-8 assay demonstrated that tumor cell killing was significantly attenuated after MHC I blockade, while MHC II blockade also partially weakened the cytotoxic effect (Figs. S9 and S10).

In summary, Ag-DCNV-STINGa not only promoted dendritic cell maturation and inflammatory factor secretion, but also effectively activated T cells and enhanced their cytotoxicity against bladder cancer cells. Compared with DCNVs or DCNV-STINGa, the engineered nanovesicles provided stronger and more stable immune activation. Besides, the immune activation and antitumor effects induced by Ag-DCNV-STINGa rely on MHC-mediated antigen presentation. These results supporting their potential as an immunoengineered platform for bladder cancer immunotherapy.

### 3.3 Safety evaluation and in vivo biodistribution of DC nanovesicles

**Fig. 4:**
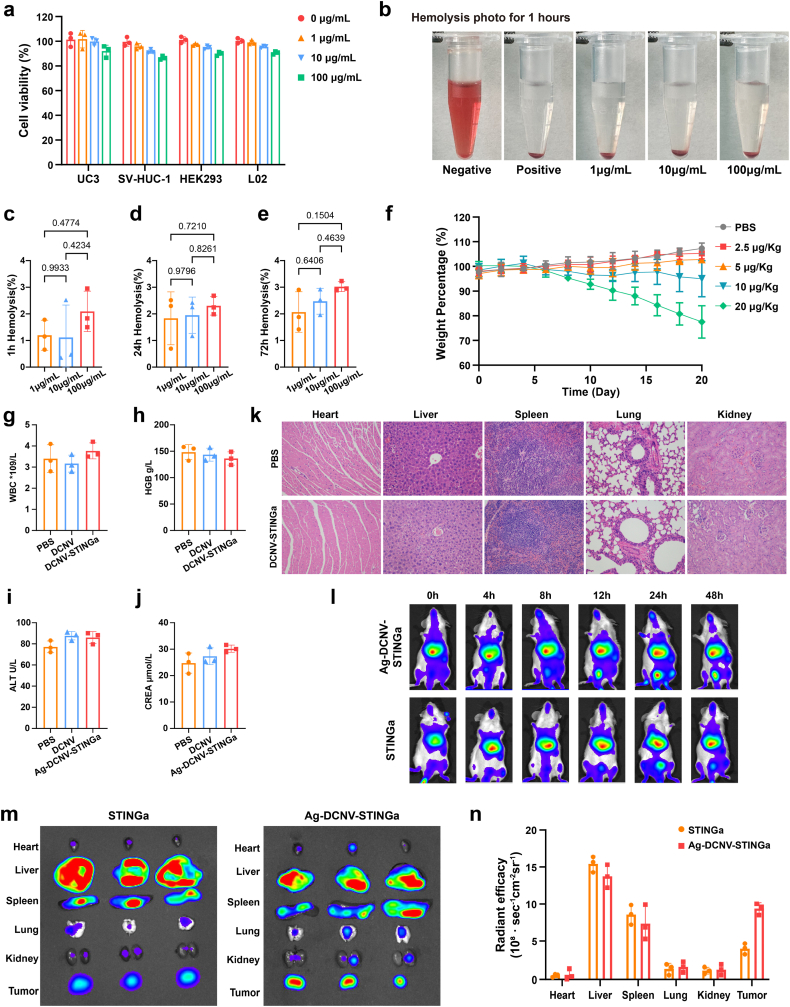
**Biosafety evaluation and in vivo biodistribution of Ag-DCNV-STINGa.** (a) In vitro direct cytotoxicity assessment. UC3 bladder cancer cells, normal bladder epithelial cells, HEK293 cells, and L02 cells were incubated with Ag-DCNV-STINGa in the absence of PBMCs, followed by CCK-8 analysis to compare cell viability. (b-e) Hemolysis assay evaluating blood compatibility. Red blood cells were incubated with Ag-DCNV-STINGa at different concentrations (1, 10, and 100 μg/mL) for 1 h, 24 h, and 72 h, followed by representative images/absorbance readings and quantitative analysis of hemolysis rate (PBS as negative control and hemolytic reagent as positive control). (f) In vivo tolerability assessment. Immunodeficient mice received multiple intravenous injections of different doses of DCNV or PBS, and body weight changes were continuously monitored. The 20 μg/kg group showed body weight loss, whereas body weight remained stable in the 10 μg/kg group; therefore, 10 μg/kg was used as the safe dose in subsequent in vivo experiments. (g-j) Hematological and biochemical analysis at the safe dose (10 μg/kg), including white blood cell count (WBC) (g), hemoglobin (HGB) (h), alanine aminotransferase (ALT) (i), and creatinine (CREA) (j), to evaluate systemic toxicity and liver/kidney function. (k) Histological evaluation of major organs. H&E staining of the heart, liver, spleen, lung, and kidney was performed to assess inflammation, necrosis, or structural damage under different treatments. (l) In vivo biodistribution by live imaging. DiR-labeled Ag-DCNV-STINGa was intravenously administered, and IVIS imaging was performed at different time points. (m,n) Ex vivo IVIS imaging of major organs at 24 h (m) and quantitative analysis of fluorescence intensity (n), showing relatively high signal in tumors together with distribution in the liver and spleen.

To assess the biocompatibility of Ag-DCNV-STINGa, we first examined its direct cytotoxicity in vitro. In the absence of PBMCs, UM-UC-3 bladder cancer cells, normal bladder epithelial cells, HEK293 cells, and normal human liver L02 cells were incubated with Ag-DCNV-STINGa and analyzed by CCK-8. Cell viability was not significantly reduced in any of these cell types. Indicating that Ag-DCNV-STINGa itself did not exert direct cytotoxicity, the observed antitumor effect depended mainly depend on immune cells (Fig. 4a). Blood compatibility was further evaluated by an in vitro hemolysis assay. At concentrations of 1, 10, and 100 μg/mL and incubation times of 1, 24, and 72 h, no obvious hemoglobin release was observed from the RBC suspension, the hemolysis rate remained extremely low. This indicate that the nanovesicles do not disrupt erythrocyte membrane integrity and display good hemocompatibility (Fig. 4b–e). In vivo safety was then evaluated in immunodeficient mice. We continuously monitered the body weight and repeated intravenous administration of different doses of DCNVs or PBS. A certain degree of weight loss was observed at 20 μg/kg, whereas body weight remained stable at 10 μg/kg. Therefore, the safety evaluation dose refers to vesicle protein amount (10 μg/kg), while the therapeutic dose is 250 μg vesicle protein per mouse per dose, corresponding to approximately 5 μg 2′3′-cGAMP equivalent per mouse (Fig. 4f). At this dose, routine blood tests and serum biochemistry showed that white blood cell counts, hemoglobin, alanine aminotransferase (ALT), creatinine (CREA), and other measured parameters remained within the normal range (Fig. 4g–j and Supplementary Figure), suggesting that liver and kidney function were not detectably affected. Histological evaluation of major organs, including the heart, liver, spleen, lung, and kidney, revealed no obvious inflammatory infiltration, necrosis, or structural damage compared with PBS controls, further demonstrating the favorable tissue tolerance of Ag-DCNV-STINGa (Fig. 4k).

To investigate the in vivo biodistribution, we labeled Ag-DCNV-STINGa with the near-infrared dye DiR and administered intravenously. Whole-body fluorescence imaging showed that a clear signal was detectable in the tumor area 24 h after injection, and this signal was stronger than that observed in non-tumor tissues and in the free STINGa solution group (Fig. 4l). Ex vivo IVIS imaging and quantitative analysis of major organs at 24 h further showed relatively high signal intensity in tumors, together with detectable distribution in the liver and spleen (Fig. 4m and n). This pattern suggests that the nanovesicles circulate through the bloodstream, enter the reticuloendothelial system, and accumulate passively in the tumor region, providing a delivery basis for subsequent immune activation. Collectively, Ag-DCNV-STINGa showed no direct cytotoxicity in vitro, exhibited good hemocompatibility, and did not cause obvious liver or kidney injury or histopathological abnormalities after repeated intravenous administration in vivo. Together with the detectable tumor accumulation and distribution in immune-related organs such as the liver and spleen, these results indicate that the engineered nanovesicles can achieve effective systemic delivery while maintaining a favorable safety profile.

### 3.4 DC nanovesicles suppress bladder cancer growth in humanized immune-reconstituted mice

**Fig. 5:**
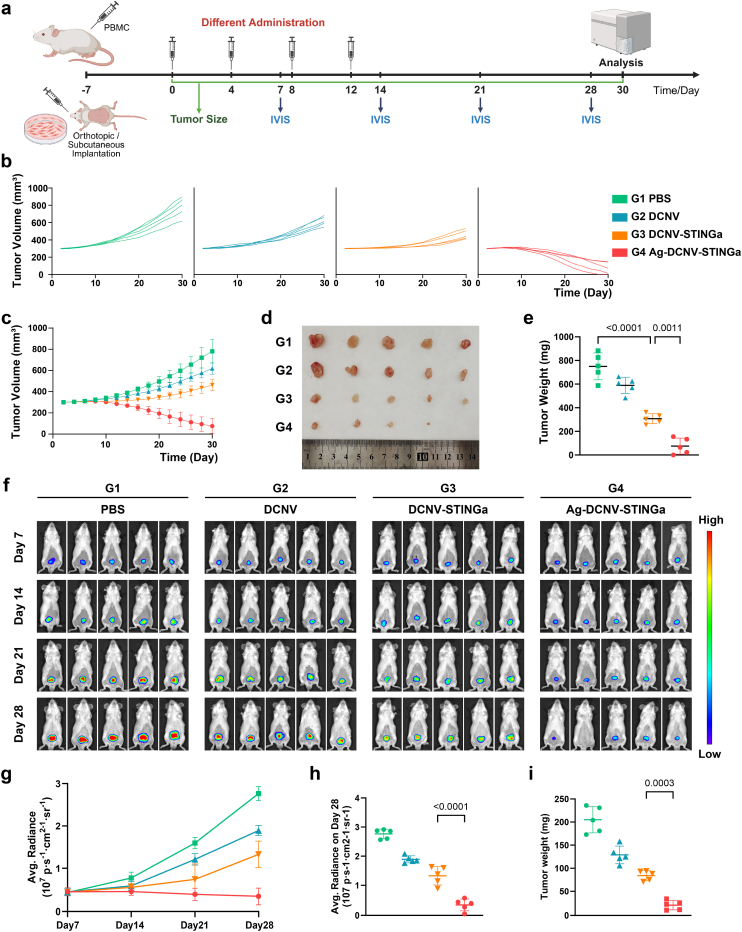
**Ag-DCNV-STINGa suppresses bladder cancer growth in Hu-PBMC-humanized NSG mice.** (a) Schematic illustration of Hu-PBMC humanization, tumor establishment, and treatment schedule. NSG mice were intravenously injected with human PBMCs for immune reconstitution while Luc-labeled bladder cancer subcutaneous or orthotopic models were established; treatment was initiated after tumor formation and administered according to a q3d × 4 schedule (once every 3 days for a total of 4 doses). (b) Schematic illustration of tumor volume measurement and calculation in the subcutaneous tumor model (tumor length and width were measured every 2 days). (c) Subcutaneous tumor growth curves showing tumor volume changes over time in PBS, empty DCNV, DCNV-STINGa, and Ag-DCNV-STINGa groups. (d,e) Final tumor weights (d) and representative photographs of excised tumors at the endpoint (e), used to evaluate the therapeutic effects on subcutaneous tumor burden. (f) IVIS monitoring schedule and fluorescence intensity curve of the orthotopic bladder cancer model during treatment. (g) Representative IVIS images of orthotopic tumors during treatment, showing changes in bioluminescence signal over time in different treatment groups. (h,i) Quantification of orthotopic tumor fluorescence intensity at the endpoint (h) and comparison of tumor weights at the endpoint (i).

To evaluate the in vivo antitumor activity of Ag-DCNV-STINGa, we first established Hu-PBMC immune-reconstituted mouse models. Human PBMCs were injected intravenously into NSG mice to generate Hu-PBMC humanized mice [27,28]. The immune reconstructed level was measured by flow cytometric analysis of mice spleen after one week (Fig. S11). T24 bladder cancer cells were implanted subcutaneously. Stable human cell engraftment and established tumors were observed 7 days later, after which treatment was initiated every 3 days for a total of four administrations; the overall experimental schedule is shown in Fig. 5a. In the T24 subcutaneous tumor model, tumor length and width were measured every 2 days and tumor growth curves were generated (Fig. 5b). Tumors in the control group grew rapidly over time. Empty DCNV modestly slowed tumor progression, and DCNV-STINGa further reduced the growth rate. In contrast, tumors in the Ag-DCNV-STINGa group grew most slowly and remained the smallest throughout the observation period (Fig. 5c). At the endpoint, tumors were excised and weighed. Compared with PBS and empty-vesicle controls, the Ag-DCNV-STINGa group showed markedly reduced tumor volume and weight, and the excised tumors were visibly smaller, indicating strong in vivo tumor suppression (Fig. 5d and e). Subcutaneous tumor formation with UM-UC-3 cells showed similar results, as presented in Fig. S12.

To further assess therapeutic efficacy, an orthotopic bladder cancer model was established in humanized NSG mice by implanting luciferase-labeled T24 cells (T24-Luc) into the bladder wall; the schedule is also shown in Fig. 5a. During treatment, tumor burden was monitored by serial IVIS imaging, and fluorescence intensity curves were generated (Fig. 5f). In the control group, tumor-associated bioluminescence continuously increased over time. Free STINGa slowed this increase to some extent, whereas Ag-DCNV-STINGa markedly reduced the signal or maintained it at a relatively low level throughout the treatment period (Fig. 5g). At the endpoint, both orthotopic tumor fluorescence intensity and tumor weight were lower in the Ag-DCNV-STINGa group than in the other groups (Fig. 5h and i), indicating that the nanovesicles were also effective in reducing tumor burden in the orthotopic setting.

**Fig. 6:**
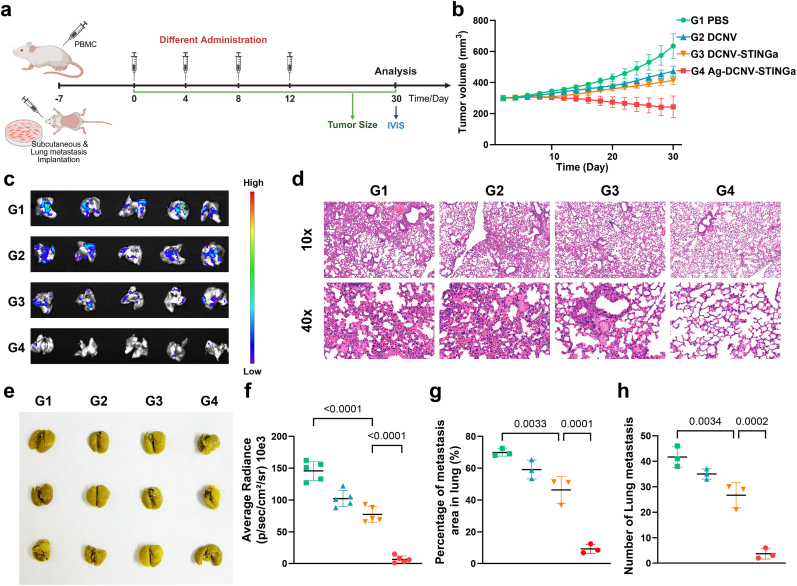
**Ag-DCNV-STINGa reduces lung metastatic burden of bladder cancer in Hu-PBMC-humanized NSG mice.** (a) Schematic illustration of the lung metastasis model and treatment schedule. While establishing subcutaneous tumors, T24-Luc bladder cancer cells were intravenously injected to induce lung metastasis, followed by Hu-PBMC humanization; treatment was then administered according to the q3d × 4 schedule. (b) Growth curve of subcutaneous primary tumors in the lung metastasis model, with tumor volume measured every 2 days to compare the inhibitory effects of different treatments on primary tumor growth. (c,f) Ex vivo IVIS imaging of lung tissues at the endpoint (c) and quantitative analysis of fluorescence signal (f), used to assess lung metastatic burden. (d,g) Representative H&E staining images of lung tissues (d) and pathological evaluation/quantification (g), comparing metastatic infiltration and tissue damage among groups. (e,h) Representative photographs of lung surface metastatic nodules after Bouin's fixation (e) and quantification of metastatic nodules (h).

To examine effects on distant metastasis, T24-Luc cells were injected intravenously to induce lung metastasis at the time of subcutaneous tumor establishment, together with humanized immune reconstruction, the schedule is shown in Fig. 6a. During treatment, subcutaneous tumor growth was recorded continuously and followed the same trend observed in the single subcutaneous model, with the Ag-DCNV-STINGa group consistently showing the lowest tumor volumes (Fig. 6b). At the endpoint, ex vivo IVIS imaging of the lungs showed obvious pulmonary fluorescence signals in the control group, while the signal was reduced in the free STINGa group and was markedly decreased in the Ag-DCNV-STINGa group (Fig. 6c,f). H&E staining showed multiple tumor nodules in the lungs of control mice, whereas only scattered small foci or no obvious tumor infiltration was observed after Ag-DCNV-STINGa treatment (Fig. 6d,g). Bouin's fixation further confirmed that the number of visible metastatic nodules on the lung surface was significantly reduced in the Ag-DCNV-STINGa group (Fig. 6e,h).

Taken together, in Hu-PBMC humanized mice, Ag-DCNV-STINGa significantly reduced tumor volume, tumor weight, and bioluminescent tumor burden in subcutaneous, orthotopic, and lung metastasis models, and also decreased the number of pulmonary metastatic nodules. Compared with DCNV or DCNV-STINGa, the Ag-DCNV-STINGa produced more stable and sustained antitumor effects in vivo, supporting their potential as an effective therapeutic platform.

### 3.5 Ag-DCNV-STINGa remodels the tumor immune microenvironment and induces tertiary lymphoid structure formation

**Fig. 7:**
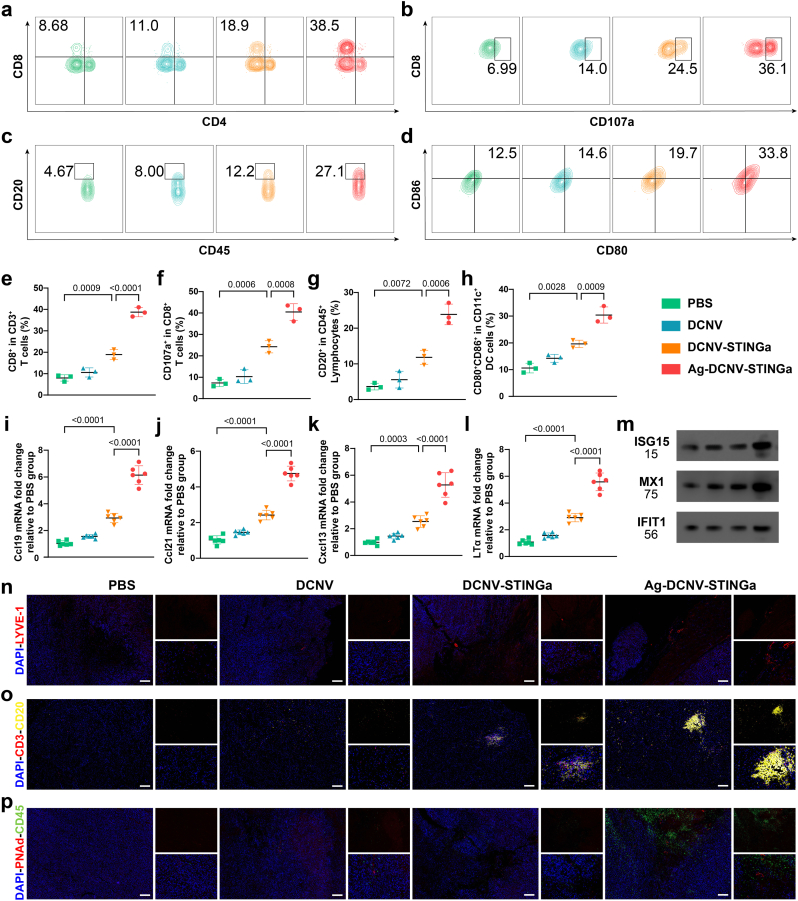
**Ag-DCNV-STINGa remodels the tumor immune microenvironment and induces tertiary lymphoid structure (TLS) formation.** (a-d) Representative flow cytometric plots of tumor tissues collected at the endpoint from the orthotopic bladder cancer model, showing the proportion of tumor-infiltrating CD8^+^ T cells (a), expression of CD8^+^ T-cell degranulation markers (such as CD107a) (b), the proportion of CD20^+^ B cells among CD45^+^ immune cells (c), and the proportion of CD80^+^CD86^+^ double-positive mature DCs among CD11c^+^ dendritic cells (d). (e-h) Quantitative analysis corresponding to (a-d): CD8^+^ T-cell proportion (e), CD8^+^ T-cell degranulation level (f), proportion of CD20^+^ cells (g), and mature DC proportion (h). (i-l) RT-qPCR analysis of chemokines/lymphoid-organizing factors in tumor tissues: CCL19 (i), CCL21 (j), CXCL3 (k), and LTα (l), used to evaluate tumor-localized immune cell recruitment and organization signals. (m) Western blot analysis of interferon-stimulated genes (ISGs) in tumor tissues, including ISG15, MX1, and IFIT1, to evaluate sustained STING-associated interferon responses. (n) Immunofluorescence (IF) staining of tumor tissues showing LYVE-1 single staining to visualize the distribution and abundance of lymphatic-like structures. (o) CD3/CD20 double immunofluorescence staining of tumor tissues, showing spatial compartmentalization of T-cell and B-cell zones. (p) PNAd/CD45 double immunofluorescence staining of tumor tissues, showing PNAd^+^ high endothelial venule (HEV)-like structures and their association with immune cell aggregation.

To further investigate the effects of Ag-DCNV-STINGa on the local tumor immune microenvironment, tumors from orthotopic bladder cancer models were collected at the endpoint and analyzed by flow cytometry. Compared with PBS and empty DCNV treatment, Ag-DCNV-STINGa significantly increased the proportion of CD8^+^ T cells in tumor tissues (Fig. 7a,e). At the same time, expression of degranulation markers in CD8^+^ T cells was increased, indicating enhanced cytotoxic function (Fig. 7b,f). The proportion of CD20^+^ cells among CD45^+^ immune cells was also clearly increased (Fig. 7c,g). In parallel, the proportion of mature CD80/CD86 double-positive dendritic cells within the CD11c^+^ population was significantly elevated, suggesting enhanced activation of antigen-presenting cells (Fig. 7d,h). To evaluate signals involved in immune cell recruitment, chemokine expression in tumor tissues was measured by RT-qPCR. The mRNA levels of CCL19, CCL21, CXCL3, and LTα were all markedly upregulated in the Ag-DCNV-STINGa group compared with the control and empty DCNV groups (Fig. 7i–l), indicating a strengthened local chemotactic program that may support immune cell recruitment and organized immune aggregation.

Tumor sections were then analyzed by immunofluorescence to examine structural changes within the tumor microenvironment. LYVE-1 single staining showed more abundant lymphatic-like structures in the treated tumors (Fig. 7n). CD3/CD20 double staining revealed clear spatial segregation of T-cell and B-cell areas (Fig. 7o). PNAd/CD45 double staining further showed the presence of PNAd^+^ high endothelial venule-like structures within the tumor (Fig. 7p). Together, these findings indicate that Ag-DCNV-STINGa treatment induced organized immune cell aggregation and the formation of tertiary lymphoid structures (TLSs)-like immune aggregates characterized by distinct T- and B-cell zones. Because phosphorylation of the STING-TBK1-IRF3 pathway is a short-lived event, whereas the present study used a q3d × 4 treatment schedule and tumors were collected approximately 10 days after the last dose, we further measured the expression of interferon-stimulated genes (ISGs) to evaluate the sustained consequences of STING activation in tumor tissues. Western blotting showed that ISG15, MX1, and IFIT1 were significantly upregulated in the Ag-DCNV-STINGa group compared with the PBS and empty-DCNV groups (Fig. 7m), indicating a sustained type I interferon–responsive state within the tumor. Ag-DCNV-STINGa treatment increased immune cell infiltration, upregulated chemokine expression, and was accompanied by enhanced type I interferon responses and TLS-like immune aggregates formation, indicating that the engineered nanovesicles induced substantial remodeling of the local tumor immune microenvironment [29,30].

## 4 Discussion

In this study, we developed an engineered nanovesicle platform based on human moDC membranes, termed Ag-DCNV-STINGa. This system combines a biomimetic membrane carrier with stable STING agonist anchoring of a STING agonist. It achieved effective immune activation, showed favorable biocompatibility, and produced significant antitumor activity in humanized bladder cancer models. Compared with empty DCNV agonist treatment, the major advantage of this platform is that it combines membrane-derived antigen presentation and co-stimulation features with controlled delivery and stronger immune activation, which may improve both therapeutic efficacy and translational potential.

From the perspective of engineering, Ag-DCNV-STINGa is not only a conventional nanocarrier. More importantly, it functions as an immunoengineered dendritic cell membrane signal-integration platform. Using a low-osmotic disruption, sonication, and gradient purification workflow [31], we prepared membrane-derived nanovesicles that preserved a closed bilayer structure. On its surface retained key immunological molecules including MHC I/II and CD80/CD86 [32]. As a result, the vesicles provided a functional interface similar to the APCs. Covalent anchoring of 2′3′-cGAMP through Sulfo-SMCC further improved the stability and effiiciency of STING delivery. In comparision, the free STINGa are often rapidly diluted in vivo and internalized inefficiently [33]. Consistent with this design logic, DCNV-mediated delivery significantly enhanced the uptake of fluorescent cGAMP by cDC1 cells, indicating that improved APC entry is a crucial upstream event for the following immune amplification [34].

In cellular levels, Ag-DCNV-STINGa induced strong immune response in the co-culture systems of PBMC and bladder cancer cells. Tumor cell viability was markedly reduced, live/dead staining showed increased cell death, and key inflammatory and chemotactic mediators, including CXCL10, IL-2, IFN-γ, and TNF-α, were significantly elevated. At the same time, the T cell activation markers CD69 and CD25 and the DC maturation markers CD80 and CD86 were also upregulated. This pattern suggests that the system did not only activate one immune cell, but also coordinated APC activation and effector T cell activation within the same microenvironment. Importantly, isolated moDCs showed increased p-TBK1 and p-IRF3 after treatment. This directly supporting that the immune effect were driven by canonical STING pathway activation rather than nonspecific stimulation from residual membrane co-stimulatory signals alone.

The in vivo results further showed that this platform retained active in more complex biological environments. In Hu-PBMC reconstituted NSG mice, Ag-DCNV-STINGa inhibited tumor progression in the all subcutaneous, orthotopic, and lung metastasis models. Tumor growth curves was suppressed, endpoint tumor weights was reduced, orthotopic IVIS signals was declined, and pulmonary metastatic burden was decreased. Similar findings across multiple disease models supports the stable of the platform. In addition, biodistribution studies showed detectable accumulation in tumors together with distribution in the liver and spleen. This pattern is consistent with the known behavior of membrane-derived nanovesicles and supports their role along the immune organ-tumor axis [35].

A important finding of this study is that Ag-DCNV-STINGa did not induce only a transient immune response. It also appeared to reshape the tumor microenvironment into a more organized and immune reactive niche. Tumors from treated mice showed increased CD8^+^ T cell infiltration, a higher proportion of CD20^+^ B cells, more mature dendritic cells. Chemokines and lymphoid-organization associated factors such as CCL19, CCL21, CXCL3, and LTα were upregulated, while ISG15, MX1, and IFIT1 remained elevated at the endpoint. These suggesting a sustained type I interferon responsive state. Together with the increased LYVE-1^+^ structures, clear CD3/CD20 zoning, and the appearance of PNAd^+^ HEV-like structures. These data support the view that Ag-DCNV-STINGa promotes local immune cell recruitment, spatial organization, and TLS formation. TLSs are increasingly linked to better responses to immunotherapy in human cancers [[36], [37], [38]]. This gives the platform a stronger translational relevance and suggests that STING activation in this context drives not only short-term inflammation but also broader remodeling of the local immune ecosystem [39,40]. The safety profile of Ag-DCNV-STINGa is also notable. Compared with free STING agonists, which may raise concerns of systemic inflammatory toxicity, vesicle-mediated delivery combined with preferential uptake by APCs may help widen the therapeutic window [41]. Compared with previously reported STING agonist delivery platforms, which primarily focus on improving pharmacokinetics or cellular uptake. The Ag-DCNV-STINGa system integrates tumor antigen priming, dendritic cell membrane co-stimulatory features, and surface-conjugated STING agonist delivery, providing a multi-functional approach to enhance antitumor immunity.

There are also several limitations of the present study. First, although covalent anchoring of 2′3′-cGAMP to the vesicle surface is achievable, more rigorous quantification of actual loading, batch-to-batch reproducibility, and its in vivo presentation or release behavior would strengthen the formulation and quality control framework. Second, while Hu-PBMC NSG mice allow evaluation of human immune cell-mediated antitumor responses, this model has important limitations, including graft-versus-host disease risk, incomplete reconstruction of myeloid and lymphoid compartments, and limited experimental window. These factors should be considered when interpreting translational relevance. Finally, as with many cell membrane-derived vesicle platforms, large scale production and manufacturing consistency remain practical challenges [42,43]. Even so, the preparation strategy used here is based on relatively mature membrane isolation and physical reassembly procedures and should be tried for further engineering optimization. Especially combined with clearly defined quality attributes such as size, membrane protein composition, STINGa loading, and endotoxin levels.

Overall, Ag-DCNV-STINGa represents an immunoengineering strategy that combines a biomimetic membrane interface with innate immune signal delivery. By stably delivering a STING agonist to APCs, activating STING signaling, amplifying T cell effector responses, and promoting local immune restructuring and TLS-like immune aggregates formation, this platform generated strong antitumor effects across several in vivo models. Given the compatibility of DC membrane vesicles with both endogenous immune molecules, the platform may also have broader adaptability beyond the present system. It may provide a useful foundation for the development of next generation immunotherapies for bladder cancer and potentially other malignancies.

## CRediT authorship contribution statement

**Xianlu Zhang:** Data curation, Formal analysis, Methodology, Writing – original draft, Writing – review & editing. **Peng Xin:** Data curation, Formal analysis, Writing – review & editing. **Yang Du:** Formal analysis, Investigation. **Ran Wang:** Writing – review & editing. **Yutao Wang:** Visualization, Writing – original draft. **Jianbin Bi:** Supervision, Writing – review & editing. **Yang Liu:** Conceptualization, Funding acquisition, Writing – review & editing.

## Declaration of competing interest

The authors declare that they have no known competing financial interests or personal relationships that could have appeared to influence the work reported in this paper.

## Data Availability

Data will be made available on request.
