# Supplementary material for: Biomimetic delivery of a STING agonist via tumor antigen-primed dendritic cell membrane nanovesicles for bladder cancer immunotherapy
Source: Mater Today Bio. 2026 Jun 11;39:103333. doi: 10.1016/j.mtbio.2026.103333 (PMC13320289; doi:10.1016/j.mtbio.2026.103333)
Supplement: Multimedia component 1 [file mmc1.docx]

**Supplementary materials**

**Biomimetic delivery of a STING agonist *via* tumor antigen-primed dendritic cell membrane nanovesicles for bladder cancer immunotherapy**

*Xianlu Zhang^a,1^, Peng Xin^a,1^, Yang Du^a,1^, Ran Wang^b,c^, Yutao Wang^d,^*, Jianbin Bi^a,^**,* *Yang Liu^a,^****

^a^ Department of Urology, The First Hospital of China Medical University, Shenyang 110001, Liaoning, China

^b^ Mater Research Institute, The University of Queensland, Brisbane, QLD4102, Australia

^c^ Institute of Chemical Biology, Shenzhen Bay Laboratory, Shenzhen 518132, China

^d^ Department of Urology, Peking Union Medical Collage Hospital, Beijing, China

^1^These authors contributed equally to this article.

*Corresponding authors.

Yutao Wang, *E*-mail: ytwang96@cmu.edu.cn

Jianbin Bi, *E*-mail: [jianbinbi@cmu.edu.cn](mailto:jianbinbi@cmu.edu.cn)

Yang Liu, *E*-mail: [urologyleo@126.com](mailto:urologyleo@126.com)


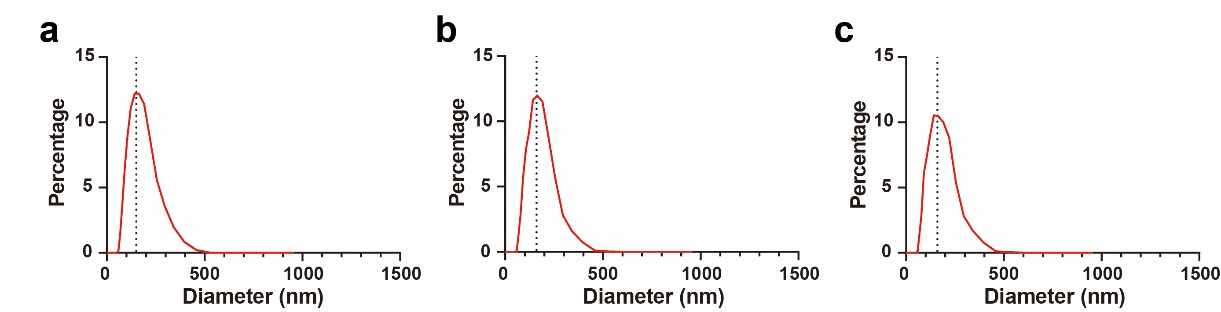


**Figure S1 Stability of Ag-DCNV-STINGa particle size under different storage conditions.** (a) Particle size distribution of freshly prepared Ag-DCNV-STINGa. (b) Particle size distribution of Ag-DCNV-STINGa after storage at −20 °C for 1 week. (c) Particle size distribution of Ag-DCNV-STINGa after storage at 4 °C for 1 week. These data were used to evaluate the stability of Ag-DCNV-STINGa under different storage conditions.


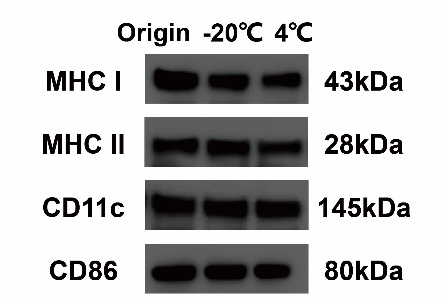


**Figure S2 Stability of membrane-associated functional proteins in Ag-DCNV-STINGa during storage.** Western blot analysis of major membrane-associated functional proteins in freshly prepared Ag-DCNV-STINGa and after storage at −20 °C or 4 °C for 1 week. No marked reduction in the expression of key membrane proteins was observed after storage, indicating that Ag-DCNV-STINGa maintained its membrane protein integrity under these conditions.


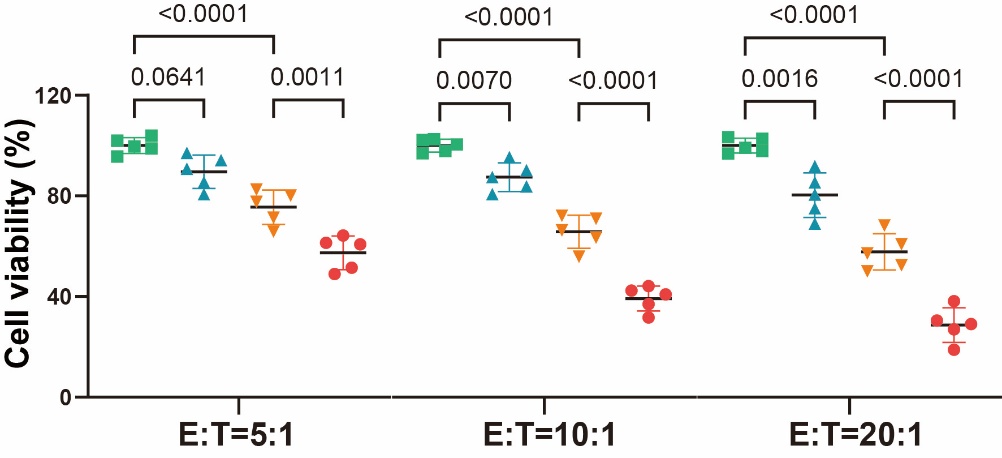


**Figure S3 CCK-8 analysis of tumor cell killing in the UM-UC-3 coculture system.** CCK-8 assay showing the viability of UM-UC-3 bladder cancer cells after coculture with PBMCs under different treatment conditions, including PBS, empty DCNVs, DCNV-STINGa, and Ag-DCNV-STINGa. These results further validate the tumor-killing effect of DCNV–STINGa in an additional bladder cancer cell line.


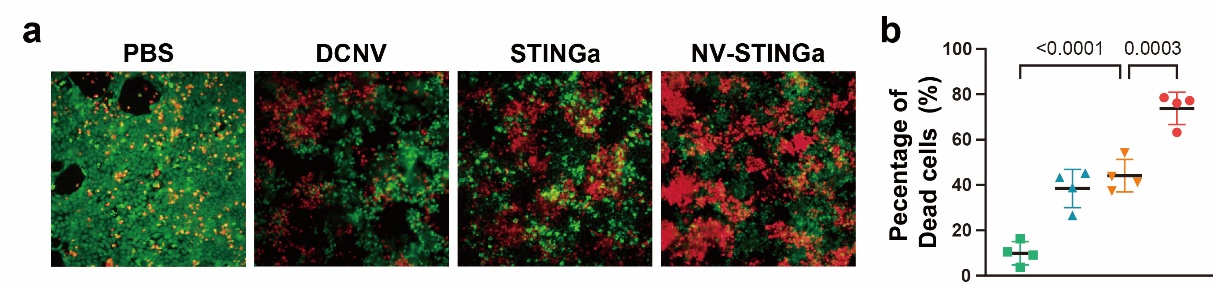


**Figure S4 Live/dead staining analysis of UM-UC-3 cells in the PBMC coculture system.** (a) Representative live/dead fluorescence staining images of UM-UC-3 cells after coculture with PBMCs under different treatment conditions. Green indicates live cells and red indicates dead cells. (b) Quantitative analysis of live/dead staining results. These data further confirm the enhanced tumor cell killing induced by Ag-DCNV-STINGa in the UM-UC-3 coculture system.


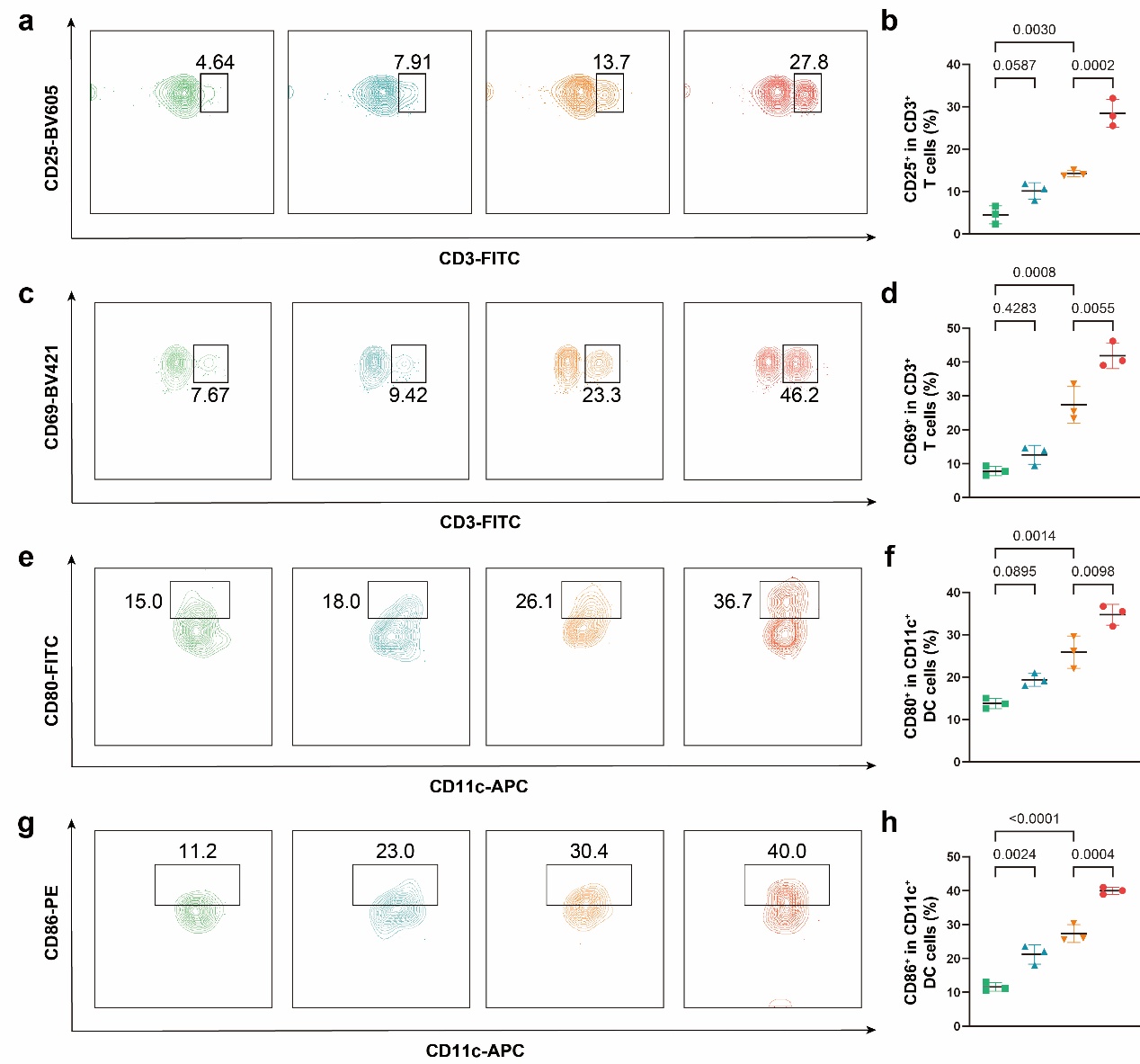


**Figure S5 Flow cytometric analysis of immune activation in the UM-UC-3/PBMC coculture system.** (a,b) Representative flow cytometric plots and quantitative analysis of CD25 expression in T cells. (c,d) Representative flow cytometric plots and quantitative analysis of CD69 expression in T cells. (e,f) Representative flow cytometric plots and quantitative analysis of CD80 expression in dendritic cells. (g,h) Representative flow cytometric plots and quantitative analysis of CD86 expression in dendritic cells. These results show that Ag-DCNV-STINGa enhances both T-cell activation and dendritic cell maturation in the UM-UC-3/PBMC coculture system.


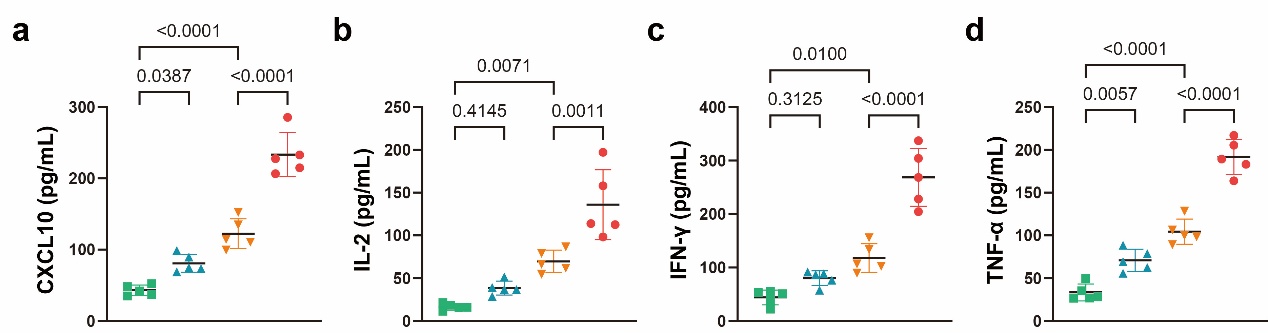


**Figure S6 ELISA analysis of cytokine and chemokine secretion in the UM-UC-3/PBMC coculture system.** (a-d) ELISA analysis of CXCL10 (a), IL-2 (b), IFN-γ (c), and TNF-α (d) in the supernatants of UM-UC-3/PBMC coculture systems under different treatment conditions. These data further support the ability of Ag-DCNV-STINGa to amplify antitumor immune activation in vitro.


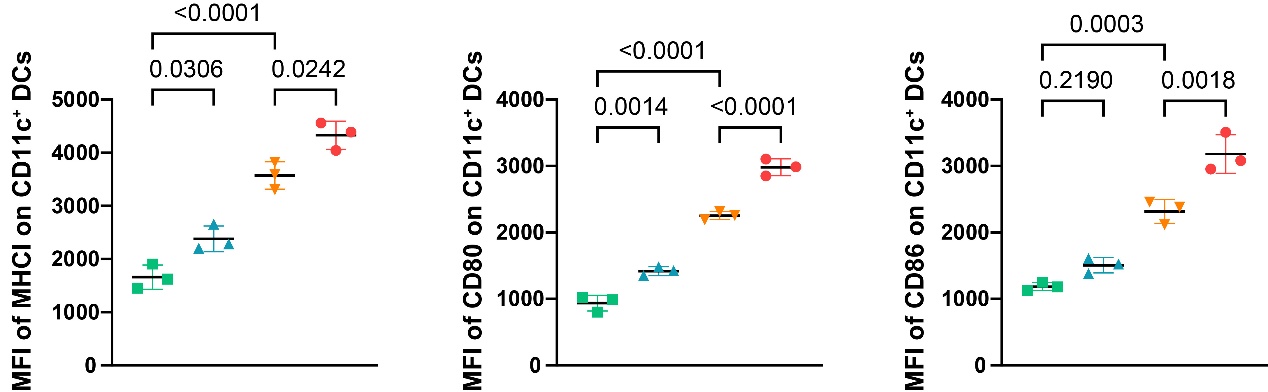


**Figure S7. Flow cytometry analysis of dendritic cell maturation markers.** Human PBMC-derived moDCs were treated with PBS, DCNV, DCNV-STINGa, or Ag-DCNV-STINGa for 24 h. The mean fluorescence intensity (MFI) of CD80, CD86, and MHC I on CD11c⁺ DCs was measured.


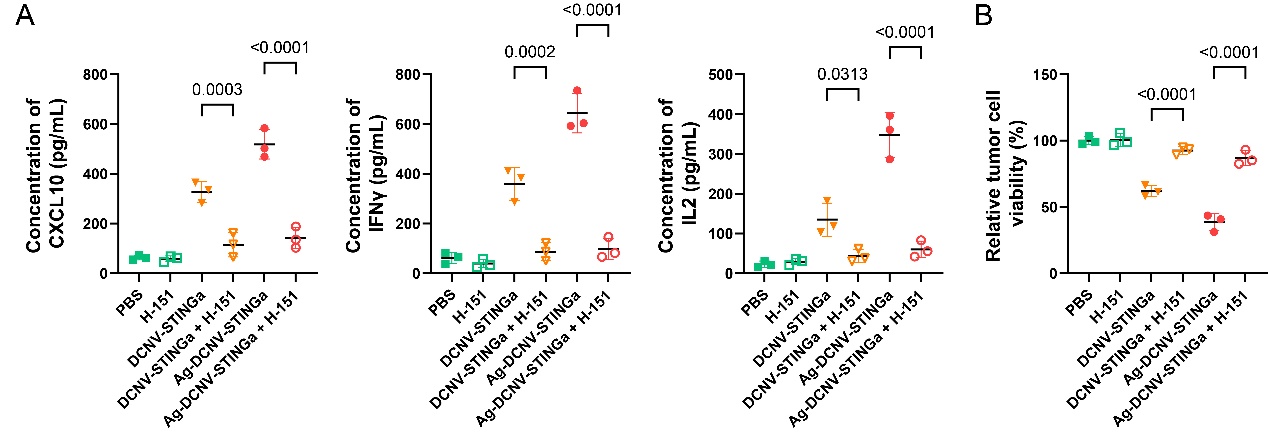


**Figure S8. STING inhibitor attenuates Ag-DCNV-STINGa-induced immune activation and tumor killing in vitro.** (A) Concentrations of CXCL10, IFN-γ, and IL-2 in PBMC–bladder cancer co-culture supernatants were measured by ELISA after treatment with PBS, H-151, DCNV-STINGa, DCNV-STINGa + H-151, Ag-DCNV-STINGa, or Ag-DCNV-STINGa + H-151 for 24 h. (B) Tumor cell viability was determined using CCK-8 assay under the same treatment conditions. Data are presented as mean ± SD from three independent biological replicates.

**
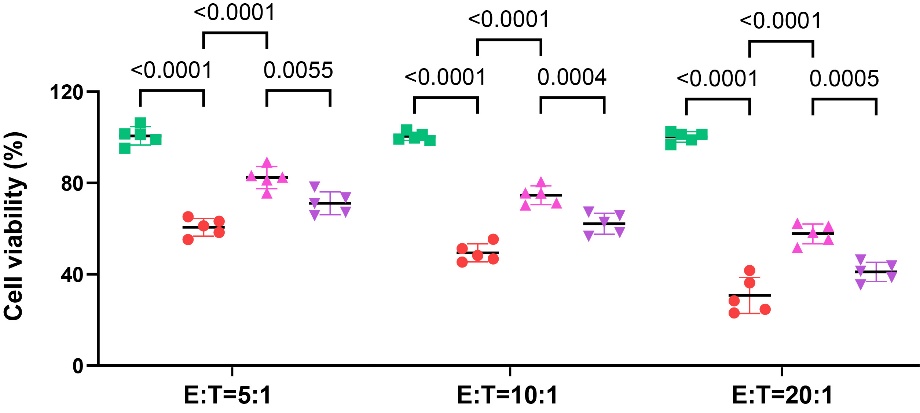
**

**Figure S9. MHC I and MHC II blocking assays: CCK-8.** In the CCK-8 assay performed at different effector-to-target (E:T) ratios, blockade of MHC I markedly reduced the cytotoxic effect induced by Ag-DCNV-STINGa. Blocking MHC II also decreased Ag-DCNV-STINGa-induced killing, although the reduction was less pronounced than that observed after MHC I blockade.


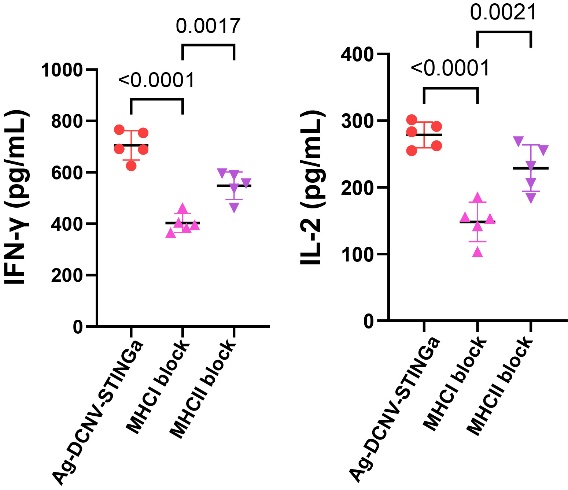


**Figure S10. MHC I and MHC II blocking assays: ELISA analysis of IFN-γ and IL-2.** Blocking MHC I markedly reduced the secretion of IL-2 and IFN-γ induced by Ag-DCNV-STINGa. Blocking MHC II also decreased Ag-DCNV-STINGa-induced cytokine secretion, but the reduction was less pronounced than that observed after MHC I blockade.


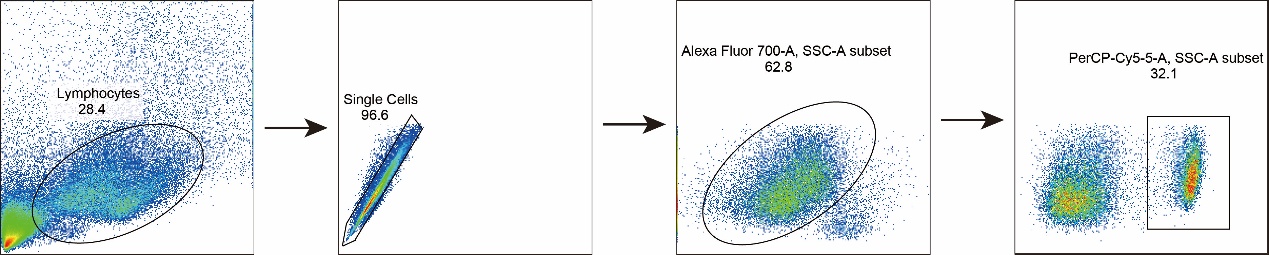


**Figure S11 Flow cytometric evaluation of human immune cell reconstitution in humanized mice.** Flow cytometric analysis of mouse spleen tissue showing that human CD45⁺ cells accounted for approximately 30% of live cells, indicating successful human immune system reconstitution in HU-PBMC NSG mice.


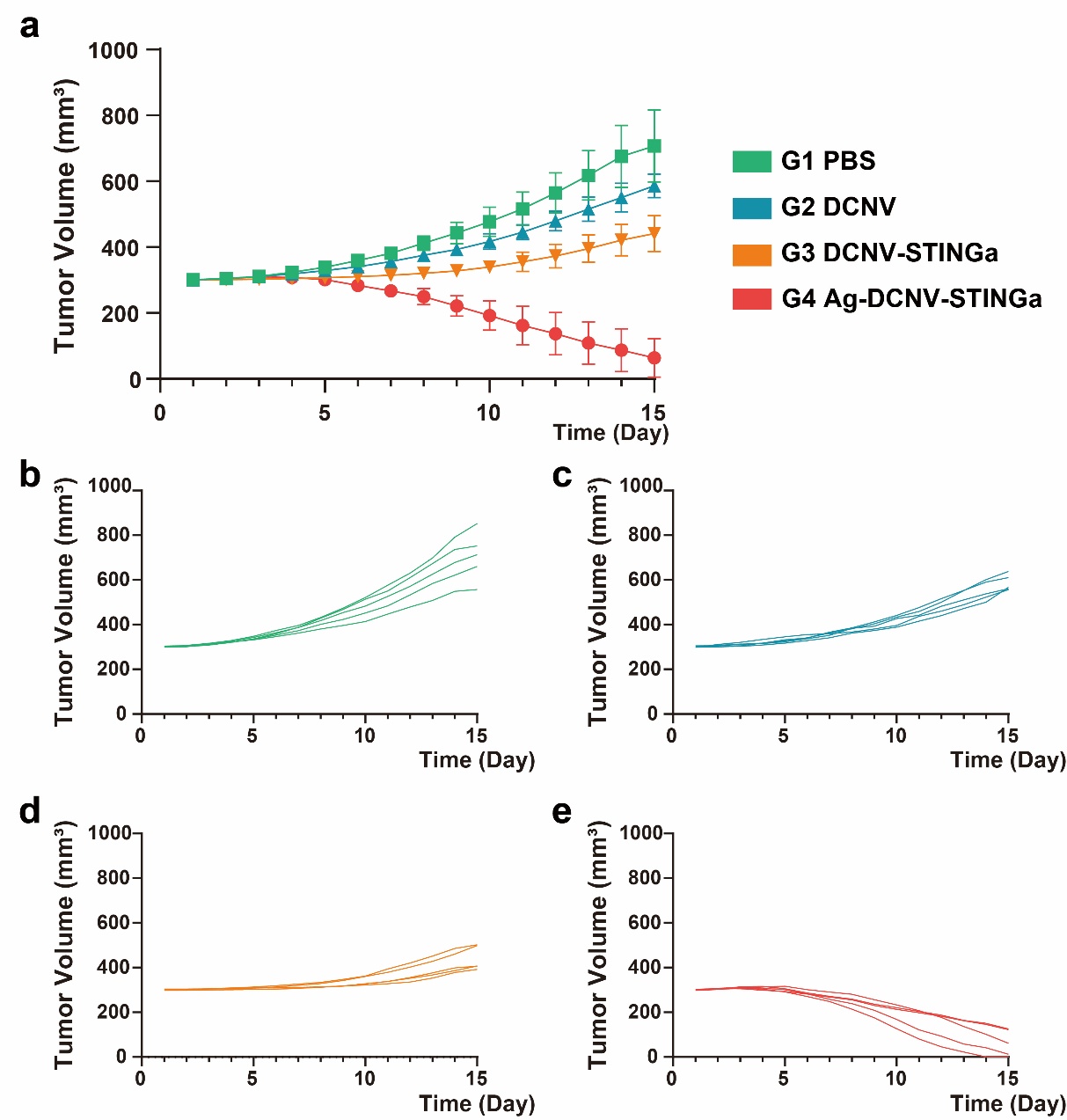


**Figure S12 Antitumor efficacy of Ag-DCNV-STINGa in the UM-UC-3 subcutaneous tumor model.** (a) Overall tumor growth curve of UM-UC-3 subcutaneous tumors under different treatment conditions. (b-e) Individual tumor growth curves in the PBS group (b), empty DCNV group (c), DCNV-STINGa group (d), and Ag-DCNV-STINGa group (e). Subcutaneous tumor formation with UM-UC-3 cells showed similar therapeutic trends to those observed in the main model.


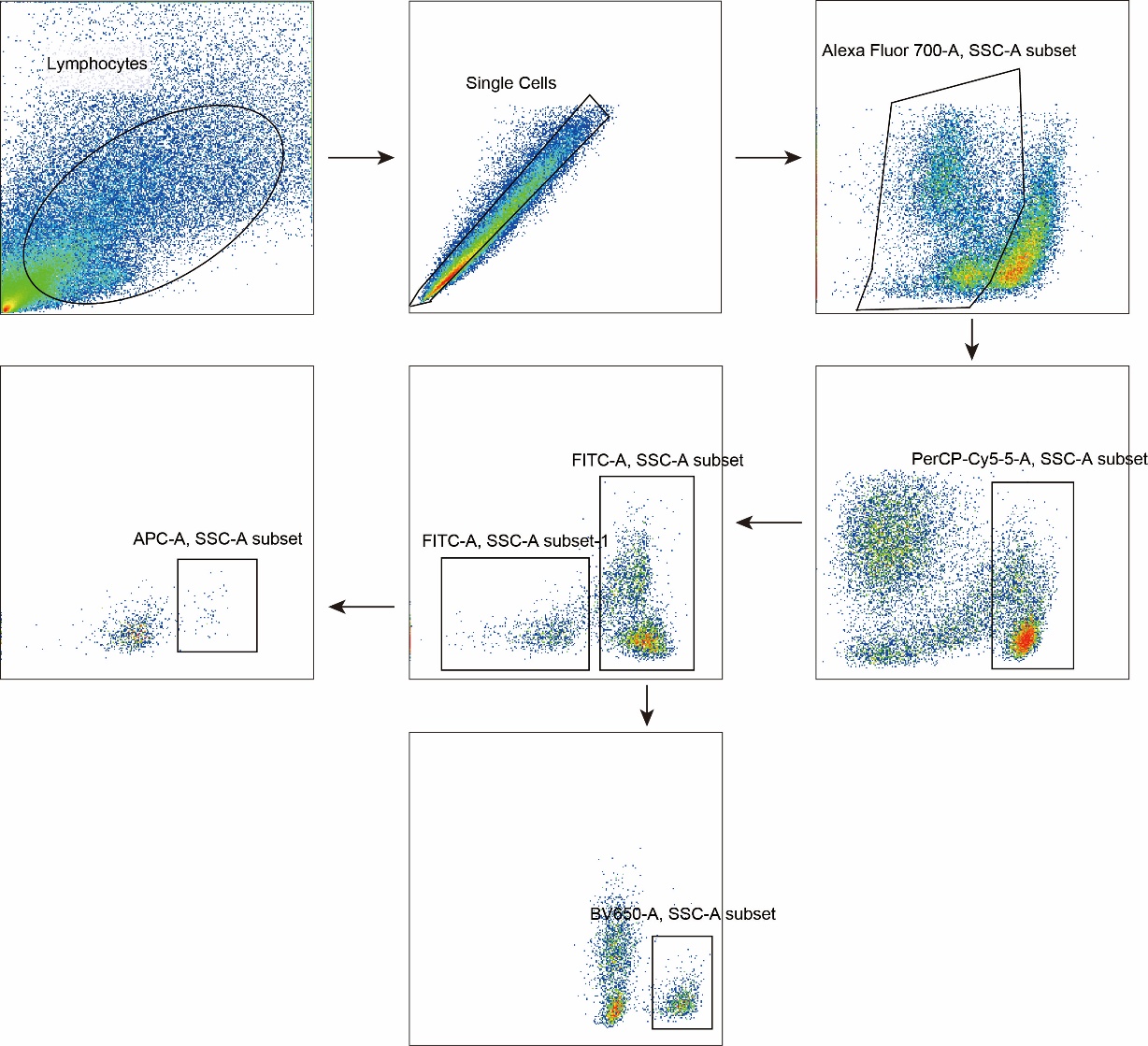


**Figure S13 Gating strategy for flow cytometric analysis.** Representative gating strategy used for flow cytometric analysis of immune cell populations in coculture systems, spleen tissues, and tumor tissues, including the identification of live cells, human CD45⁺ immune cells, T-cell subsets, dendritic cell populations, and other indicated cell groups.
